# A wheat MYB transcription factor activates defense by repressing *TaSIZ1* in response to branched-chain amino acid accumulation

**DOI:** 10.1093/plcell/koag135

**Published:** 2026-05-06

**Authors:** Swathy Puthanvila Surendrababu, Loizos Savva, Andrey V Korolev, Lionel Hill, Gerhard Saalbach, Diane G O Saunders

**Affiliations:** John Innes Centre, Norwich Research Park, Norwich, United Kingdom; John Innes Centre, Norwich Research Park, Norwich, United Kingdom; John Innes Centre, Norwich Research Park, Norwich, United Kingdom; John Innes Centre, Norwich Research Park, Norwich, United Kingdom; John Innes Centre, Norwich Research Park, Norwich, United Kingdom; John Innes Centre, Norwich Research Park, Norwich, United Kingdom

## Abstract

Branched-chain amino acid (BCAA) accumulation has been linked to the induction of salicylic acid (SA)–related defense responses in wheat (*Triticum aestivum*). Here, we explored whether the underlying mechanism might involve a wheat ortholog of SAP AND MIZ1 DOMAIN-CONTAINING LIGASE1 (SIZ1), a critical SA regulator in other systems. We found that *TaSIZ1* was significantly repressed in mutant plants disrupted in the BCAA aminotransferase gene *TaBCAT1*; these plants accumulate enhanced levels of BCAAs and SA. Overexpressing *TaSIZ1* suppressed SA-hyperaccumulation in *TaBCAT1* mutants, and treating wild-type plants with the BCAA Leu repressed *TaSIZ1* expression. Thus, *TaSIZ1* is responsive to BCAA levels and influences SA accumulation, consistent with a role linking BCAA and SA in defense. Nuclear proteomic analysis of the *TaBCAT1* mutant identified transcriptional regulators that could be modifying BCAA-responsive *TaSIZ1* expression. This included an ortholog of the *Arabidopsis thaliana* trihelix transcription repressor 6b-INTERACTING PROTEIN-LIKE1 (ASIL1) with a potential binding site in the *TaSIZ1* promoter. Further work showed that TaASIL1 bound to the *TaSIZ1* promoter. Also, disrupting *TaASIL1* function inhibited Leu-dependent *TaSIZ1* repression. Based on these data, we propose that elevated BCAA levels, such as those arising during pathogen attack, activate TaASIL1, which represses *TaSIZ1*, thereby promoting SA accumulation and SA-mediated defense in wheat.

## Teaser

The MYB transcription factor TaASIL1 links branched-chain amino acid accumulation to activation of salicylic acid-mediated defense responses via *TaSIZ1* repression in wheat.

## Introduction

Sophisticated signal transduction networks protect plants against microbial infection (Khan et al. 2022). Interlinked immune recognition systems activate pattern-triggered immunity and effector-triggered immunity, both of which depend on the phytohormone salicylic acid (SA; 2-hydroxybenzoic acid) and trigger systemic acquired resistance (SAR) or “defense priming” (Vlot et al. 2021). At the site of pathogen infection, SA rapidly accumulates, which is biosynthesized principally in chloroplasts via the isochorismate synthase pathway, rather than the alternative phenylalanine ammonia-lyase (PAL) pathway (Lefevere et al. 2020). This SA accumulation leads to extensive transcriptional reprogramming that induces SAR (Spoel and Dong 2024). During SAR, mobile chemical signals that are generated in the infected tissue are transmitted systemically to distal uninfected parts of the plant and protect them against secondary infection (Vlot et al. 2021). These signals include specialized non-proteogenic amino acids such as aminobutyric acid (BABA) (Luna et al. 2014), pipecolic acid (Pip) (Návarová et al. 2012), and its SAR bio-active derivative N-hydroxypipecolic acid (NHP; Hartmann et al. 2018). SA itself can also act as a signal transducer that is transported to distal uninfected cells via the apoplast (Lim et al. 2016). Although SA concentration largely determines the SA-mediated deployment of immune responses, the mechanisms regulating SA levels remain poorly understood (Huang et al. 2018).

Maintaining SA homeostasis is critical given that its roles in plant growth, metabolism, and abiotic stress responses (Lefevere et al. 2020) create an SA-dependent trade-off between plant growth and immunity (Zhong et al. 2021). The levels of bioactive SA can be fine-tuned via post-translational modification through glucosylation or methylation to form SA 2-O-β-D-glucoside and methyl salicylate, respectively (Dempsey et al. 2011). In the model dicot *Arabidopsis thaliana*, the glucosyltransferase UGT76B1 acts as a hub regulating SAR through the glucosylation of SA, N-hydroxypipecolic acid (NHP), and isoleucic acid [ILA, a branched-chain amino acid (BCAA)-related 2-hydroxycarboxylic acid]. Knockout mutants of UGT76B1 have elevated SA, NHP, and ILA levels and display enhanced immunity, whereas overexpressing UGT76B1 reduces free NHP and SA levels and compromises basal immunity and SAR in response to *Pseudomonas syringae* pv. *tomato* (*Pto*) infection (Bauer et al. 2021). These discoveries shed light on some central regulators of SA homeostasis, but it is not clear whether an analogous mechanism is conserved across plant species.

Successful plant pathogens hijack plant metabolism and nutrient biosynthesis to enrich nutrient availability (Divon and Fluhr 2007). However, exploiting host physiology creates changes in metabolites that plants can sense and interpret as indicators of pathogen infection (Moormann et al. 2022). This includes changes in the abundance of amino acids, which act not only as major nitrogen sources for the invading pathogen but also as critical regulators of immune signaling. For instance, BCAAs (Ile, Leu, and Val) represent energy sources for bacterial and fungal pathogens (Kaiser and Heinrichs 2018; Garbe and Vylkova 2019), yet BCAA accumulation in plants also increases SA levels, which play a central role in directing immune signaling against biotrophic pathogens (Dempsey et al. 2011). In bread wheat (*Triticum aestivum*), infection by the biotrophic yellow rust pathogen (*Puccinia striiformis* f. sp. *tritici*; *Pst*) upregulates the gene for BCAA AMINOTRANSFERASE 1 (*TaBCAT1*), which modulates catabolism of BCAAs (Ile, Leu, and Val). Notably, disrupting *TaBCAT1* leads to accumulation of BCAAs and SA, resulting in reduced susceptibility to *Pst* and the related stem rust pathogen *Puccinia graminis* f. sp. *tritici* (*Pgt*) (Corredor-Moreno et al. 2021). In rice (*Oryza sativa*), certain beneficial microbial genera reduce the expression of a BCAA aminotransferase gene, increasing BCAA levels and resistance to the rice false smut biotrophic pathogen *Ustilaginoidea virens* (Liu et al. 2023). Thus, current data point to BCAA metabolism as a key target for both detrimental and beneficial microbes to modulate SA-mediated defense responses.

To investigate the unexplored link between BCAA metabolism and SA-mediated defence responses in wheat, we focused on the SUMO E3 ligase SAP AND MIZ1 DOMAIN CONTAINING LIGASE1 (SIZ1), a key regulator of SA homeostasis in Arabidopsis (Lee et al. 2007). In Arabidopsis, AtSIZ1 acts alongside E1-activating and E2-conjugating enzymes to efficiently conjugate SUMO to the Lys residue(s) of target proteins via a process termed SUMOylation (Park et al. 2011). Arabidopsis knockout mutants of *AtSIZ1* accumulate SA and exhibit constitutive SAR, with enhanced resistance to *Pto* (Lee et al. 2007). Here, we identified a wheat SIZ1 ortholog (TaSIZ1) and determined that it is a critical regulator linking elevated BCAA levels to SA accumulation. We also identified orthologs of 2 trihelix transcription factors (TFs) with binding sites in the *TaSIZ1* promoter: ARABIDOPSIS 6B-INTERACTING PROTEIN-LIKE 1 (ASIL1) and ASIL2, termed TaASIL1 and TaASIL2, respectively. In Arabidopsis, ASIL1 and ASIL2 are closely related trihelix TFs that act as transcriptional repressors (Gao et al. 2009) during abiotic and biotic stress responses (Ruiz et al. 2021). TaASIL1, and not TaASIL2, bound to the *TaSIZ1* promoter and mediated Leu-dependent repression of *TaSIZ1* expression. Furthermore, we found that TaASIL2 function is required to maintain BCAA homeostasis and consequently acts to indirectly regulate TaASIL1-mediated suppression of *TaSIZ1* expression. Together, our findings support a model in which elevated BCAA levels activate the TF TaASIL1, which represses *TaSIZ1* expression, thereby releasing the TaSIZ1-mediated suppression of SA levels and promoting SA-mediated defense responses. The discovery of this TaASIL1–*TaSIZ1* module mediating BCAA-dependent SA regulation in wheat presents an important target for manipulation to enhance wheat resilience against rust fungi.

## Results

### Suppressing *TaSIZ1* expression increases free SA levels and pathogenicity-related (*PR*) gene expression in the *TaBCAT1* disruption mutant

**Figure 1:**
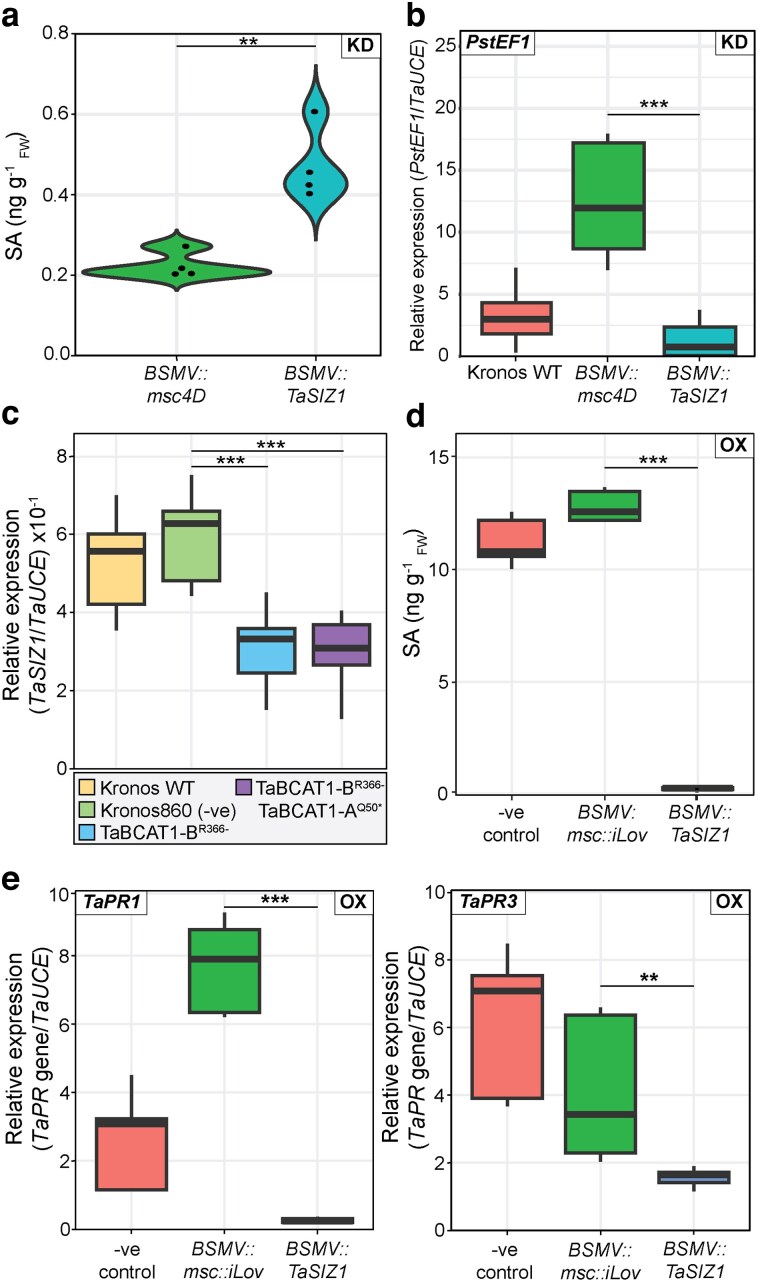
*TaSIZ1* negatively regulates salicylic acid levels (SA) in wheat and is downregulated in the *TaBCAT1* disruption mutant. a) Silencing of *TaSIZ1* increases free SA levels in wild-type (WT) tetraploid wheat plants (cv. Kronos). Plants were silenced with *BSMV::TaSIZ1* (*n* = 4) and compared to the *BSMV::msc4D* negative viral infection control (*n* = 4) at 8 d post-viral inoculation (dpvi). b) *TaSIZ1* silencing led to a significant reduction in *Pst* fungal biomass. Plants were inoculated with *BSMV::TaSIZ1* (*n* = 3) or *BSMV::msc4D* (*n* = 3), and at 14 dpvi, infected with *Pst.* RT-qPCR analysis was used to quantify *Pst* fungal biomass at 4 d post-inoculation (dpi) with *Pst* by assessing the relative expression of the *PstEF1* gene, normalized to the *TaUCE* reference gene. c) RT-qPCR revealing the significant downregulation of *TaSIZ1* in the *TaBCAT1* single (TaBCAT1-B^R366−^) and double (TaBCAT1-A^Q50*^ TaBCAT1-B^R366−^) mutant lines compared to a Kronos ethyl methanesulfonate mutant (Kronos860) carrying WT alleles of *TaBCAT1*. Kronos WT, Kronos860, TaBCAT1-B^R366−^, and TaBCAT1-A^Q50*^ TaBCAT1-B^R366−^  *n* = 3. d-e). Overexpressing *TaSIZ1* in the *TaBCAT1* double disruption mutant (TaBCAT1-A^Q50*^ TaBCAT1-B^R366−^) significantly decreases free SA levels (d) and the expression of the pathogenicity-related (*PR*) genes *TaPR1* and *TaPR3* (e). RT-qPCR was performed 8 to 10 dpvi on plants inoculated with *BSMV::TaSIZ1* (*n* = 3) and compared to the *BSMV::msc::iLov* positive fluorescent protein control (*n* = 3). Asterisks denote statistically significant differences between each pair of conditions (***: *P* < 0.001, **: *P* < 0.01; two-tailed *t*-test). KD, silencing; OX, overexpression. Box plots: bar represents median value, box signifies the upper (Q3) and lower (Q1) quartiles, and whiskers are located at 1.5 times the interquartile range.

To explore the reported link between BCAA metabolism and SA-mediated defense responses in wheat, we first searched for wheat orthologs of the SUMO E3 ligase SIZ1, a key regulator of SA homeostasis in Arabidopsis (Lee et al. 2007). We identified a predicted wheat SIZ1 ortholog (termed TaSIZ1) that is annotated as a SUMO E3 ligase (Bolser et al. 2016) and shares 52 to 53% sequence identity with AtSIZ1. To test whether TaSIZ1 regulates SA accumulation in wheat, we performed virus-induced gene silencing (VIGS) of *TaSIZ1.* We inoculated wild-type (WT) tetraploid wheat (cv. Kronos) plants with the barley stripe mosaic virus (BSMV) expressing a 216-bp fragment of *TaSIZ1* that is conserved among the two *TaSIZ1* homoeologs (Figure S1a). *BSMV::TaPDS*-mediated silencing of wheat *PHYTOENE DESATURASE* was used independently as a positive control, and *BSMV::msc4D*, which contains an artificial sequence not present in wheat, was used as a viral infection control (Lee et al. 2015). At 8 d post-viral inoculation (dpvi), the efficiency of *TaSIZ1* silencing was confirmed by RT-qPCR, with a significant decrease (log_2_ fold change of −5.61) in *TaSIZ1* expression (Figure S1b). SA levels were also significantly higher (log_2_ fold change 1.06; 0.47 ng g^−1^  _FW_ ± 0.09) in the *TaSIZ1*-silenced plants than in the control (0.22 ng g^−1^  _FW_ ± 0.03) (Fig. 1a). To examine the consequences of *TaSIZ1* silencing on *Pst* disease progression, we conducted seedling infection assays at 14 dpvi. At 4 d post-infection (dpi) with *Pst*, we employed RT-qPCR analysis to quantify relative *Pst* fungal biomass based on the relative expression of *PstEF1* normalized to the expression levels of the wheat reference gene *TaUCE* (Borrill et al. 2016; Tao et al. 2020). Compared to the control, significantly less *Pst* fungal biomass accumulated following *TaSIZ1* silencing (log_2_ fold change of −4.57) (Fig. 1b). Furthermore, there was no evidence of *Pst* pustule development at 14 dpi in *TaSIZ1*-silenced plants (Figure S1c). These data show that, similar to the role of AtSIZ1 in Arabidopsis (Lee et al. 2007), TaSIZ1 plays a key role in regulating SA-mediated defense responses in wheat.

To determine whether SIZ1 function could be linked to BCAA-mediated SA regulation, we analyzed *TaSIZ1* expression in Kronos Targeting Induced Local Lesions in Genomes (TILLING) mutants disrupted in *BCAA AMINOTRANSFERASE 1* (*TaBCAT1*), which exhibit constitutively enhanced BCAA and SA levels (Corredor-Moreno et al. 2021). In 2-week-old seedlings, *TaSIZ1* was significantly downregulated in the *TaBCAT1* single (TaBCAT1-B^R366−^) and double (TaBCAT1-A^Q50*^ TaBCAT1-B^R366−^) mutants, with a log_2_ fold change of −0.97 and −1.04, respectively, compared to the negative control: an ethyl methanesulfonate mutant in the Kronos background (Kronos860) carrying WT alleles of *TaBCAT1* (Fig. 1c). Thus, suggesting *TaSIZ1* suppression could be critical in supporting the elevation of SA seen in the *TaBCAT1* disruption mutants.

To confirm whether the reduction in *TaSIZ1* expression in the *TaBCAT1* disruption mutant is required to increase SA accumulation and *PR* gene expression, we conducted virus-induced overexpression (VOX) of *TaSIZ1* in wheat plants with disrupted *TaBCAT1* function. We cloned the full-length (2,538-bp) *TaSIZ1-A* gene in the pCassRZ-BSMVy-yb2A-Lic vector (Kanyuka 2022) and performed BSMV-mediated VOX on the *TaBCAT1* double mutant (TaBCAT1-A^Q50*^ TaBCAT1-B^R366−^) using *BSMV::msc::iLov* as a positive fluorescent protein control (Lee et al. 2015). At 8 to 10 dpvi, RT-qPCR confirmed successful *TaSIZ1* overexpression, with a strong increase (log_2_ fold change of 4.20) in *TaSIZ1* expression compared to the control (Figure S1d). Following VOX of *TaSIZ1-A*, SA levels dropped significantly, with a log_2_ fold change of −5.66 (0.28 ng g^−1^  _FW_ ± 0.16) in the VOX line compared to the control (12.10 ng g^−1^  _FW_ ± 1.82) (Fig. 1d). Furthermore, RT-qPCR revealed significant reductions in *PR1* and *PR3* expression, with *PR1* showing a log_2_ fold decrease of −4.88 and *PR3* a log_2_ fold decrease of −1.39 in the VOX line compared to the control (Fig. 1e). Thus, suppressed *TaSIZ1* expression in the *TaBCAT1* disruption mutant increased SA accumulation and *PR* gene expression, whereas overexpressing this gene had the opposite effects. Together, these findings establish *TaSIZ1* as a negative regulator of SA accumulation and *PR* gene expression in wheat.

### TaSIZ1 displays a similar domain architecture to SIZ1 orthologs from diverse plant species

**Figure 2:**
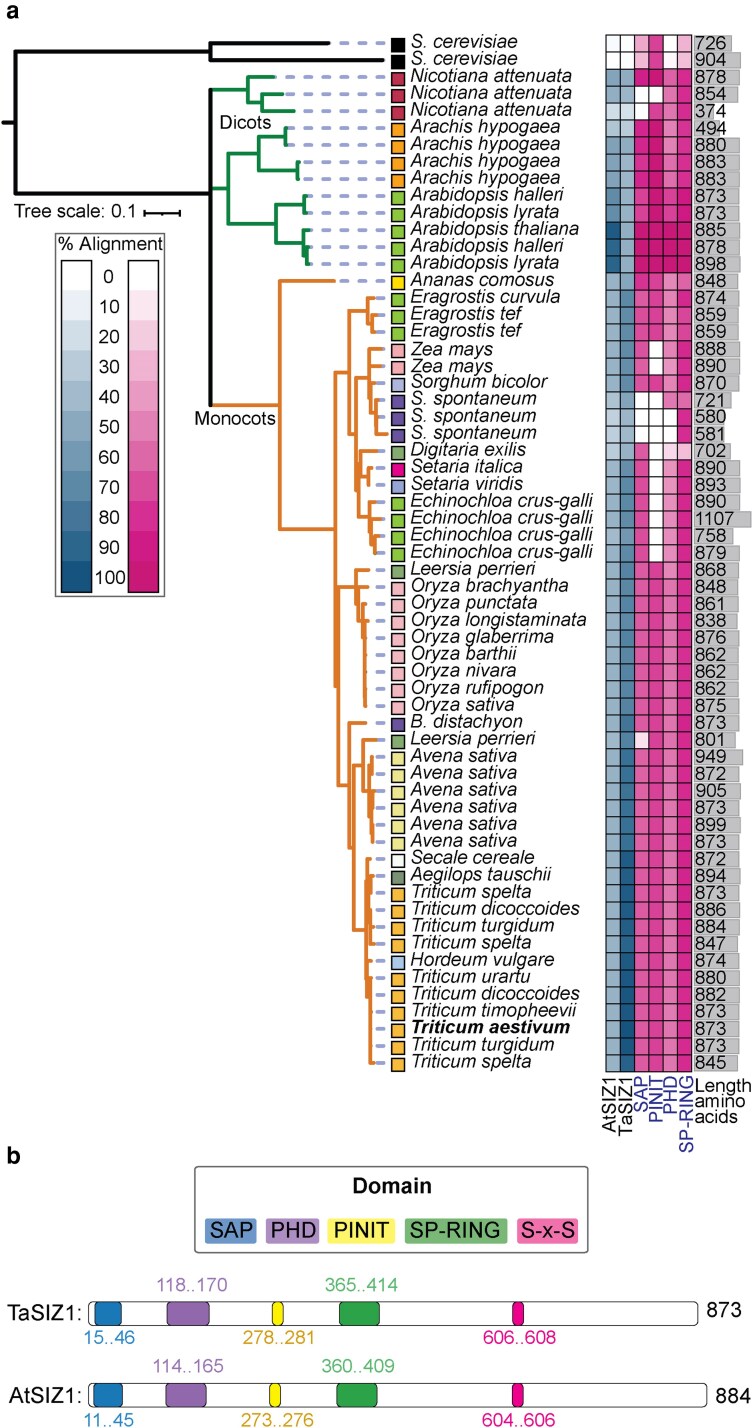
SIZ1 orthologs are present in diverse plant species, with TaSIZ1 displaying the typical modular domain architecture characteristic of AtSIZ1. a) Phylogenetic analysis of TaSIZ1 with SIZ1 orthologs from 18 plant species, indicating that TaSIZ1 shares the highest similarity with SIZ1 orthologs from species in the *Triticum* genus, *Hordeum vulgare*, and *Secale cereale*. b) TaSIZ1 contains the 5 structural elements reported in the AtSIZ1 sequence: (i) a putative N-terminal Scaffold Attachment Factor A/B, Acinus and PIAS (SAP) DNA-binding domain, (ii) a zinc finger PHD (plant homeodomain), (iii) a PINIT (Pro-Ile-Asn-Ile-Thr) motif, (iv) a central SP-RING (Siz/PIAS-RING) domain, and (v) a C-terminal S-X-S motif. Species abbreviations: *Saccharomyces cerevisiae*, *Saccharum spontaneum*, and *Brachypodium distachyon*.

A conserved domain structure is a defining characteristic of the PIAS/SIZ protein family, including SIZ1, and specific domains have been shown to be important for plant responses to different environmental stresses (Cheong et al. 2009). To investigate the level of sequence conservation between TaSIZ1 and SIZ1 orthologs in other plant species, we performed phylogenetic analysis of TaSIZ1 with SIZ1 proteins from 18 plant species (>50% identity); 2 yeast (*Saccharomyces cerevisiae*) SIZ1 sequences were used as an outgroup (Supplemental Data Set S1). The 61 SIZ1 proteins were classified into 2 groups corresponding to the 2 major angiosperm classes, dicotyledons and monocotyledons, which is consistent with previous findings (Datta et al. 2018). Despite this divergence, SIZ1 proteins in both plant classes had similar average lengths: 878 amino acids (S.D. 175) for dicotyledons and 873 amino acids (S.D. 81) for monocotyledons (Fig. 2a). Among the 18 plant species analyzed, TaSIZ1 shared the highest similarity with SIZ1 orthologs from *Triticum* species, barley (*Hordeum vulgare*), and rye (*Secale cereale*; 92.8 to 99.7% identity), while AtSIZ1 shared the highest similarity with SIZ1 orthologs in other Arabidopsis species (67.7 to 97.0% identity) (Fig. 2a).

Both TaSIZ1 and AtSIZ1 exhibited a typical modular architecture, with 5 conserved structural elements: (i) a putative N-terminal SAP (Scaffold Attachment Factor A/B, Acinus, and PIAS) DNA-binding domain essential for nuclear targeting, (ii) a zinc finger PHD (plant homeodomain) thought to be required for transcriptional repression (Miura et al. 2020), (iii) a PINIT (Pro-Ile-Asn-Ile-Thr) motif required for SUMO conjugation and nuclear retention (Cheong et al. 2010), (iv) a central SP-RING (Siz/PIAS-RING) domain crucial for SUMO E3 ligase activity (Miura et al. 2020), and (v) a C-terminal S-X-S motif required for SUMO binding (Cheong et al. 2010) (Fig. 2b and Figure S2). Across the 18 plant species, the SP-RING domain exhibited the highest sequence conservation (79.7% identity, S.D. 13.4%), followed by the SAP domain (62.3% identity, S.D. 23.1%) and the PHD homeodomain (51.7% identity, S.D. 18.4%) (Fig. 2a). Therefore, TaSIZ1 displays the typical domain architecture found among SIZ1 orthologs, which is highly conserved across a diverse array of plant species.

### *TaSIZ1* expression is modulated in response to Leu treatment

**Figure 3:**
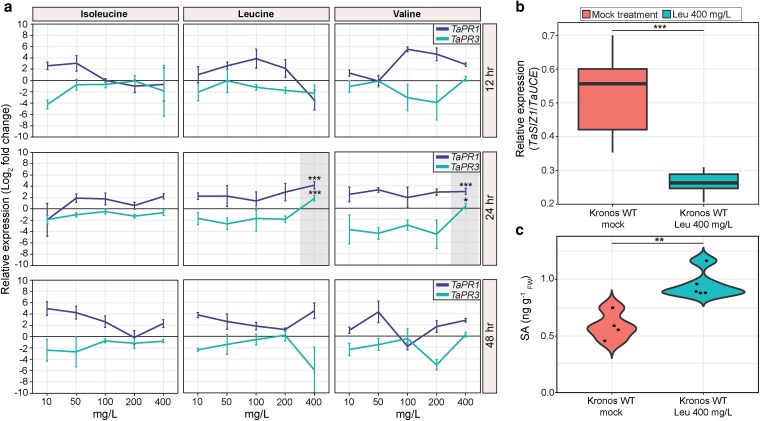
Leu treatment modulates pathogenicity-related (*PR*) gene expression, *TaSIZ1* expression, and free salicylic acid (SA) levels in wheat. a) Two wheat *PR* genes (*TaPR1* and *TaPR3*) were significantly upregulated 24 h post-treatment with 400 mg/L of the BCAAs Leu or Val, with Leu treatment having the greatest effects. RT-qPCR was performed at 12 h (*n* = 3), 24 h (*n* = 4), and 48 h (*n* = 3) after treatment with the BCAAs Ile, Leu, or Val at 10, 50, 100, 200, and 400 mg/L. Shaded areas, treatments that significantly upregulated both *TaPR1* and *TaPR3* expression compared to mock treatment; error bars, standard deviation. Asterisks only shown for conditions with statistically significant differences in both *PR1* and *PR3* expression (***: *P* < 0.001, *: *P* < 0.05; two-tailed *t*-test). b) Leu-treated samples also displayed significantly reduced *TaSIZ1* expression 24 h post-treatment (*n* = 3). c) This reduction was accompanied by a significant increase in free SA levels compared to mock-treated plants. Mock treatment *n* = 4, 400 mg/L Leu treatment *n* = 5. Asterisks denote statistically significant differences between mock and BCAA treatment (***: *P* < 0.001, **: *P* < 0.01; two-tailed *t*-test). Box plots: bar represents median value, box signifies the upper (Q3) and lower (Q1) quartiles, and whiskers are located at 1.5 times the interquartile range.

To investigate whether the repressed *TaSIZ1* expression and elevated SA-mediated *PR* expression are associated with the enhanced BCAA levels observed in the *TaBCAT1* double mutant (TaBCAT1-A^Q50*^ TaBCAT1-B^R366−^) (Corredor-Moreno et al. 2021), we evaluated *PR1*, *PR3*, and *TaSIZ1* expression in WT plants following BCAA treatment. We focused on *PR1* and *PR3* expression, as both genes have been reported to be upregulated following SA treatment in wheat (Zhang et al. 2018) and were also significantly induced in the *TaBCAT1* double disruption mutant, when compared to WT plants (Corredor-Moreno et al. 2021). We treated the leaves of WT (cv. Kronos) plants with Leu, Ile, or Val independently across a series of concentrations (10, 50, 100, 200, and 400 mg/L) and performed RT-qPCR at 0, 12, 24, and 48 h post-treatment to examine *PR1*, *PR3*, and *TaSIZ1* expression compared to mock-treated plants. All 3 compounds increased *PR1* expression at almost all concentrations and time points post-treatment compared to mock-treated control plants (Fig. 3a). However, *PR3* expression was only significantly elevated following Leu or Val treatment at 24 h post-treatment (log_2_ fold increase of 1.86 and 0.38, respectively) compared to mock-treated control plants (Fig. 3a). In addition to displaying the most significant increases in *PR1* and *PR3* expression, the Leu-treated plants also had significantly decreased *TaSIZ1* expression (log_2_ fold of −1.02) (Fig. 3b) and elevated SA levels (log_2_ fold increase of 0.69; 0.95 ng g^−1^  _FW_ ± 0.12) compared to mock-treated control plants (0.59 ng g^−1^  _FW_ ± 0.12) (Fig. 3c). In contrast, no significant change in *TaSIZ1* expression was found in Val-treated plants at 24 h post-treatment (Figure S3a). We also expanded the analysis to include a series of earlier timepoints (3, 6, 9, and 12 h) post Leu and Val treatment (400 mg/L) and found no significant changes in *PR1*, *PR3,* and *TaSIZ1* expression at these timepoints (Figure S3b-c). We conclude that *TaSIZ1* expression can be repressed 24 h post Leu treatment, in turn leading to increased *PR* gene expression and SA levels.

### MYB TFs are enriched in *TaBCAT1* disruption mutants

**Figure 4:**
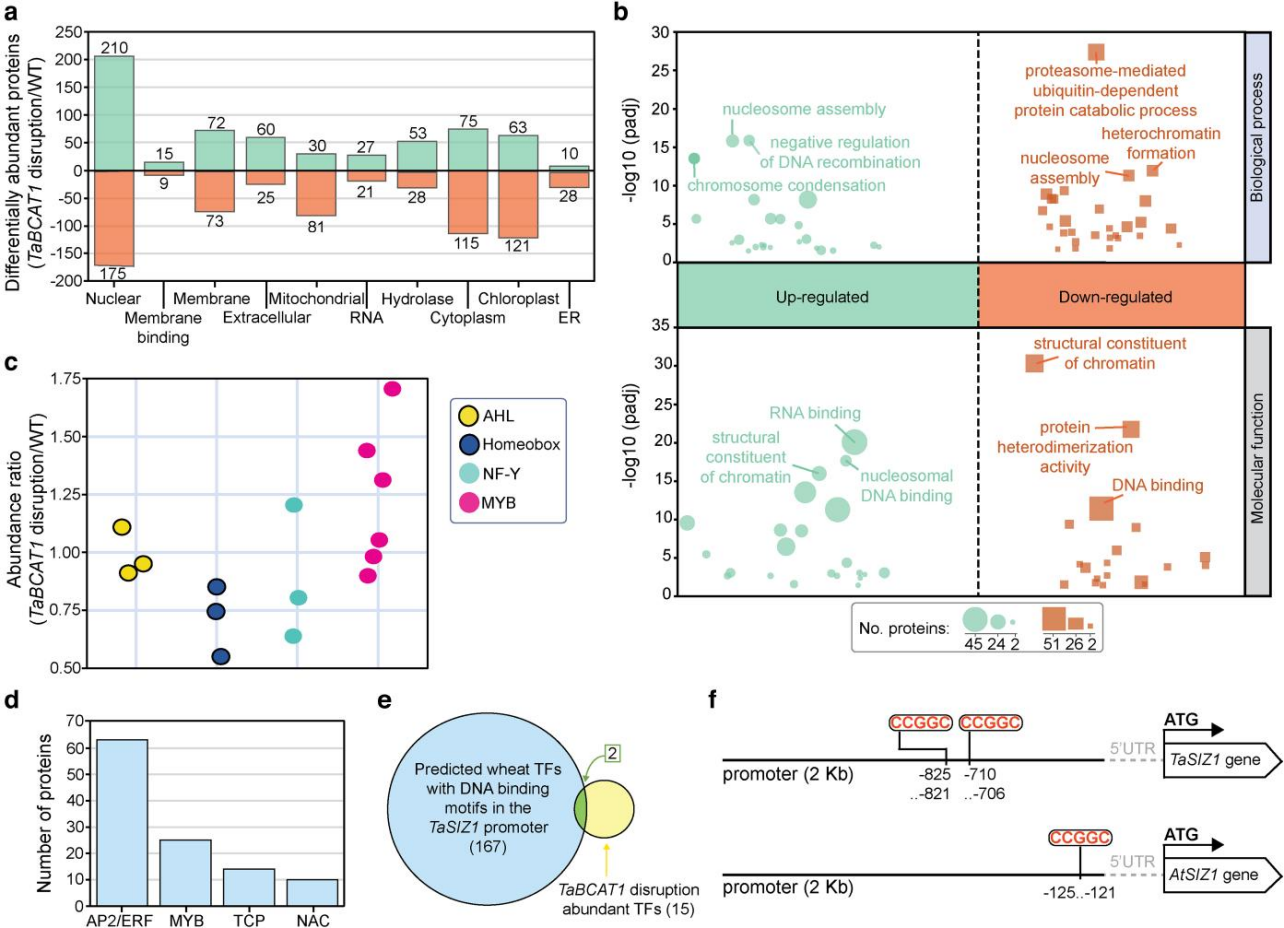
TaASIL1 and TaASIL2 are enriched in the *TaBCAT1* disruption mutant and have DNA-binding sites in the *TaSIZ1* promoter. a) Comparative nuclear proteomic analysis of the *TaBCAT1* double disruption mutant (TaBCAT1-A^Q50*^ TaBCAT1-B^R366−^; *n* = 4) and wild-type (WT) (cv. Kronos; *n* = 4) lines identified 1,291 differentially abundant proteins (DAPs) (*P-*value < 0.05). b) Results of gene ontology (GO) enrichment analysis of the 385 nuclear DAPs. padj, *P*-value adjusted. c) This analysis identified 15 proteins with potential DNA-binding function; the most abundant was the MYB (myeloblastosis viral oncogene homolog) TF family. d) A total of 498 potential DNA-binding motifs were identified in the *TaSIZ1* promoter corresponding to 167 wheat proteins that were predicted to encode TFs assigned to the following families: (i) AP2/ERF (APETALA2/ethylene-responsive factor); (ii) MYB, with a high proportion in the MYB/SANT TF subclass; (iii) TEOSINTE BRANCHED 1/CYCLOIDEA/PROLIFERATING CELL FACTOR (TCP); and (iv) NAC (NAM, ATAF, and CUC). e) Among the 167 wheat proteins predicted to bind to sites in the *TaSIZ1* promoter, two orthologs of the MYB/SANT TFs termed ASIL1 and ASIL2 were also enriched in the *TaBCAT1* double mutant compared to the WT. f) Two predicted ASIL1/2 TF binding sites (5′-CCGGC-3′) were identified in the *TaSIZ1* promoter and one in the AtSIZ1 promoter region.

To search for transcriptional regulators that could suppress *TaSIZ1* expression in the *TaBCAT1* double mutant, we performed nuclear proteomic analysis of double mutant (TaBCAT1-A^Q50*^ TaBCAT1-B^R366−^) and WT (cv. Kronos) plants. We extracted nuclei from 14-day-old seedlings and subjected the digested nuclei pellets to nano-scale liquid chromatography coupled to tandem mass spectrometry (nanoLC-MS/MS). The significantly differentiated peptides in the TaBCAT1-A^Q50*^ TaBCAT1-B^R366−^ and WT lines were assigned to 4,164 wheat proteins across all samples, with 1,291 classified as differentially abundant proteins (DAPs) (*P-*value < 0.05) in the *TaBCAT1* double mutant compared to the WT (Supplemental Data Set S2). Among the DAPs, 385 proteins (29.82%) were classified as nuclear proteins, 190 as cytoplasmic proteins (14.72%), 184 as chloroplast proteins (14.26%), 145 as membrane proteins (11.23%), 111 as mitochondrial proteins (8.60%), 85 as extracellular proteins (6.58%), 81 as hydrolase proteins (6.27%), 48 as cytoplasmic RNA binding proteins (3.72%), 38 as endoplasmic reticulum proteins (2.94%), and 24 as membrane binding proteins (1.86%). Among the 385 nuclear DAPs, 210 were enriched and 175 were depleted in the *TaBCAT1* double mutant compared to the WT (Fig. 4a and Supplemental Data Set S2).

To explore the potential functions of these 385 nuclear DAPs, we performed Gene Ontology (GO) enrichment analysis. Among GO terms in the biological processes and molecular function categories, proteins highly enriched in the *TaBCAT1* disruption mutant included DNA and RNA binding proteins and those involved in nucleosome and chromosome organization, regulation of DNA recombination, and maintaining the structural integrity of chromatin. Among nuclear proteins most depleted in the *TaBCAT1* disruption mutant were those involved in heterochromatin formation, ubiquitin-dependent protein catabolism, DNA binding, maintaining the structural integrity of chromatin, and protein heterodimerization (Fig. 4b). Further exploration identified 15 nuclear proteins with DNA-binding functions, including the following: (i) AT-HOOK MOTIF NUCLEAR LOCALIZED (AHL) TF family proteins (3 proteins), which play critical roles in plant development and biotic and abiotic stress responses (Martinčová and Soukup 2025); (ii) Homeobox and DDT domain–containing transcriptional regulators (3 proteins), which are required to maintain vegetative growth (Li et al. 2012); (iii) Nuclear factor-Y (NF-Y) TFs (3 proteins), which regulate cellular processes and stress responses (Bhattacharjee and Hallan 2023); and (iv) MYB (myeloblastosis viral oncogene homolog) TFs (6 proteins), which function in an array of plant processes (Wu et al. 2023) (Fig. 4c). Among the most abundant MYB TF family members, 4 were from the MYB/SANT TF subclass, which are crucial regulators of plant development and stress responses (Biswas et al. 2023) (Table S1).

### The MYB/SANT TFs TaASIL1 and TaASIL2 are predicted to bind to the *TaSIZ1* promoter

To investigate whether any of the 15 nuclear proteins with DNA-binding function bind to the promoter of *TaSIZ1* and regulate its expression, we analyzed the 2-kb predicted promoter sequence upstream of *TaSIZ1* for potential TF binding motifs. We identified 498 potential binding motifs through searches against the Arabidopsis motif database (Gupta et al. 2007) (Supplemental Data Set S3), which corresponded to 167 wheat genes that were predicted to encode TFs through subsequent sequence similarity searches. These core DNA-binding elements correspond to TF binding sites associated with proteins assigned to (i) the AP2/ERF (APETALA2/ethylene-responsive factor) family (63 proteins), which regulate plant responses to abiotic stress (Ma et al. 2024); (ii) the MYB family (25 proteins), with a high proportion (22 proteins) assigned to the MYB/SANT TF subclass; (iii) the TEOSINTE BRANCHED 1/CYCLOIDEA/PROLIFERATING CELL FACTOR (TCP) family (14 proteins), which regulate plant organogenesis and morphogenesis (Wang et al. 2025); and (iv) the NAC (NAM, ATAF, and CUC) (10 proteins) family, which play crucial roles in plant growth, development, and stress responses (Yuan et al. 2019) (Fig. 4d and Supplemental Data Set S4). Among the 167 predicted wheat TFs with potential DNA-binding sites in the *TaSIZ1* promoter, 2 proteins corresponding to orthologs of 2 predicted MYB/SANT TFs (TraesCS3B02G305100 and TraesCS6B02G230000) were also enriched in the nuclear proteome of the *TaBCAT1* mutant compared to the WT (Fig. 4e). Sequence similarity searches indicated that these 2 potential TFs are likely orthologs of AtASIL1 and AtASIL2, which are trihelix members of the MYB/SANT family. Analysis of cis-regulatory motifs in the *TaSIZ1* promoter identified a predicted AtASIL1 TF binding site (5′-CCGGC-3′) that was also present in the *AtSIZ1* promoter sequence (Fig. 4f and Supplemental Data Set S5), pointing to a possible conserved cross-species role for ASIL1 and/or ASIL2 in modulating *SIZ1* expression.

**Figure 5:**
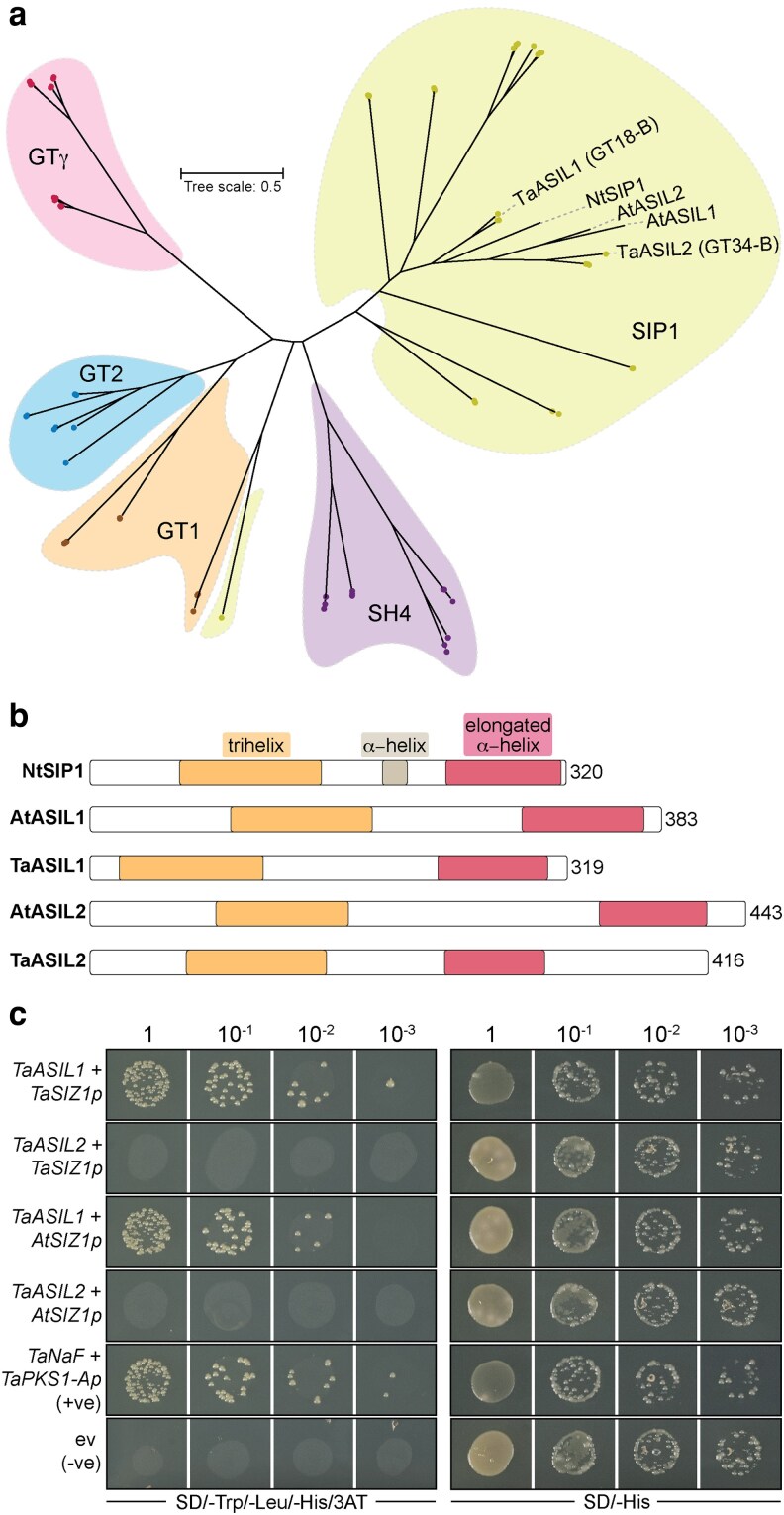
The predicted TaASIL1 and TaASIL2 orthologs belong to the SIP1 clade, harbor conserved trihelix and α-helical domains, typical of SIP clade members, and TaASIL1 binds to the *TaSIZ1* promoter. a) TaASIL1 and TaASIL2 were grouped in the SIP1 clade in phylogenetic analysis conducted with 80 previously defined wheat trihelix transcription factors (TFs) (also known as GT-factors). The SIP1 clade member from tobacco (*Nicotiana tabacum*; NtSIP1) was included for comparison. b) TaASIL1 and TaASIL2 harbor the same conserved trihelix and α-helical domains, typical of SIP1 TF clade members. c) AtASIL1, and not AtASIL2, bound to the *TaSIZ1* and *AtSIZ1* promoter regions in yeast one-hybrid assays. Co-transformed yeast (Y187) strains were assessed for growth after 7 to 10 d of incubation. All transformed strains grew well on SD/−His medium, confirming successful transformation, and only strains carrying pHIS2-TaSIZ1 + pGADT7-TaASIL1, pHIS2-AtSIZ1 + pGADT7-TaASIL1, or the positive control pHIS2-TaPSK1-A + pGADT7-TaNF-YB1 exhibited robust growth on restrictive medium (SD/−Trp/−Leu/−His/3 − AT).

To classify the predicted TaASIL1 and TaASIL2 sequences within the 5 distinct trihelix TF clades previously defined in Arabidopsis (SIP1, GT-1, GT-2, GTγ, and SH4) (Yasmeen et al. 2016), we conducted phylogenetic analysis using 80 previously defined wheat trihelix TFs (Wang et al. 2019) (also known as GT-factors) as well as AtASIL1 and AtASIL2. As AtASIL1 and AtASIL2 belong to the SIP1 clade (Yasmeen et al. 2016), we also included the SIP1 clade member from tobacco (*Nicotiana tabacum*), NtSIP1 (Wang et al. 2019). This analysis grouped TaASIL1 and TaASIL2 within the SIP1 clade (Fig. 5a); these proteins harbor conserved trihelix and α-helical domains typical of SIP1 clade members (Fig. 5b). Therefore, TaASIL1 and TaASIL2 share high sequence conservation with their Arabidopsis counterparts. This finding, along with the conservation of the ASIL1 binding site in the wheat and Arabidopsis *SIZ1* promoters, suggests that they may also target similar genes.

### TaASIL1 binds to the *TaSIZ1* and *AtSIZ1* promoters in yeast one-hybrid assays

To determine whether TaASIL1 and/or TaASIL2 directly regulate *TaSIZ1* expression, we performed a yeast one-hybrid assay. We amplified a 2-kb DNA fragment immediately upstream of the 5′UTR of *TaSIZ1* and fused it to the minimal *HIS3* promoter in the pHIS2 vector. Similarly, due to conservation in the predicted ASIL1/2 binding site between the *TaSIZ1* and *AtSIZ1* promoters, we also cloned the 2-kb promoter region of Arabidopsis *AtSIZ1* into pHIS2 for comparison. In parallel, we cloned the full-length coding sequences (CDS) of *TaASIL1* (1,879 bp) and *TaASIL2* (2,375 bp) into the yeast expression vector pGADT7. As a positive control, we included the promoter of a wheat phytosulfokine gene (*TaPSK1-A*) in pHIS2 and TaNF-YB1 in pGADT7, which interacted in a previous yeast one-hybrid screen (Zhang et al. 2024). We co-transformed yeast competent cells (strain Y187) with the combined vectors, and after 7 to 10 d of incubation, assessed the yeast cells for growth. All co-transformed strains grew well on SD/-His medium, confirming successful transformation (Fig. 5c). However, on restrictive medium (SD/-Trp/-Leu/-His/3AT), strains expressing *TaASIL1* with *TaSIZ1* or *AtSIZ1* exhibited robust growth, whereas strains carrying *TaASIL2* with *TaSIZ1* or *AtSIZ1* failed to grow (Fig. 5c). Therefore, TaASIL1, and not TaASIL2, interacts with promoter sequences of both *TaSIZ1* and *AtSIZ1*.

### *TaASIL1* is required to maintain suppressed *TaSIZ1* expression in the *TaBCAT1* disruption mutant

**Figure 6:**
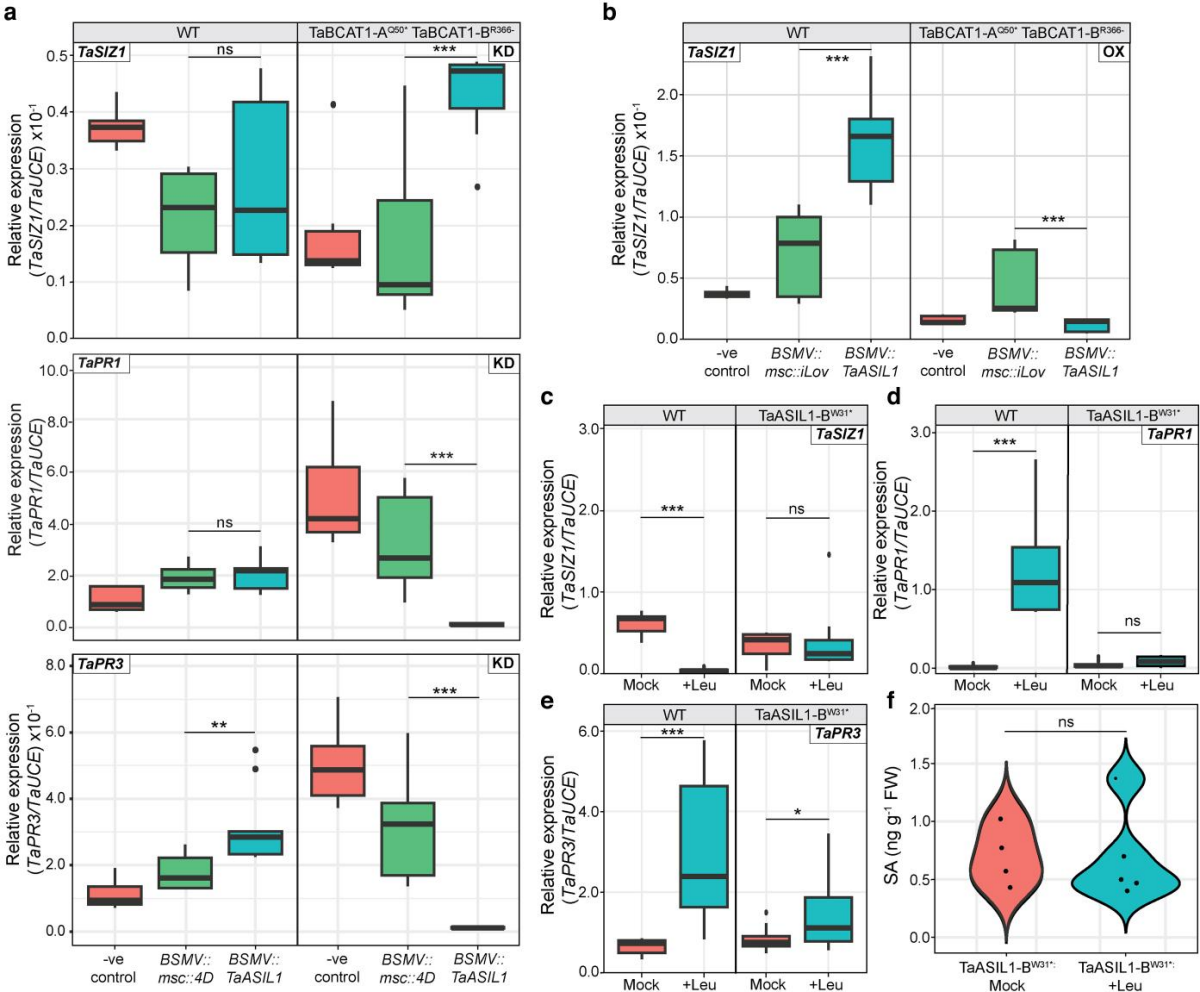
TaASIL1 function is required to maintain repressed *TaSIZ1* expression and elevated *TaPR1* and *TaPR3* expression in the *TaBCAT1* disruption mutant and following leu treatment. a) *TaSIZ1* was significantly upregulated, and *TaPR1* and *TaPR3* were downregulated following silencing of *TaASIL1* in the *TaBCAT1* double disruption mutant (TaBCAT1-A^Q50*^ TaBCAT1-B^R366−^). Plants were silenced with *BSMV::TaASIL1* (*n* = 3) and compared to the *BSMV::msc4D* negative viral infection control (*n* = 3) at 7 to 10 d post-viral inoculation (dpvi). b) Overexpression of *TaASIL1* in the *TaBCAT1* double mutant led to a significant reduction in *TaSIZ1* expression, whereas WT plants displayed a significant increase in *TaSIZ1* expression. RT-qPCR was performed 8 to 10 dpvi on plants inoculated with *BSMV::TaASIL1* (*n* = 3) and compared to the *BSMV::msc::iLov* positive fluorescent protein control (*n* = 3). c-e) The *TaASIL1* disruption mutant (TaASIL1-B^W31*^) displayed no change in *TaSIZ1* expression following Leu treatment and only minor increases in *TaPR1* and *TaPR3* expression. By contrast, wild-type (WT) plants displayed a significant reduction in *TaSIZ1* expression and increased *TaPR1* and *TaPR3* expression following Leu treatment. f) The *TaASIL1* disruption mutant (TaASIL1-B^W31*^) also displayed no change in free salicylic acid (SA) accumulation in response to Leu treatment. Asterisks denote statistically significant differences between each pair of conditions (***: *P* < 0.001, **: *P* < 0.01; *: *P* < 0.05; 2-tailed *t*-test). KD, silencing; OX, overexpression. Bar represents median value, box signifies the upper (Q3) and lower (Q1) quartiles, and whiskers are located at 1.5 times the interquartile range.

Following confirmation of a physical interaction between TaASIL1 and the *TaSIZ1* promoter, we explored whether TaASIL1 is required for *TaSIZ1*-mediated regulation of SA defense signaling. We performed VIGS to silence *TaASIL1* independently alongside *BSMV::TaPDS* and *BSMV::msc4D* in the *TaBCAT1* double mutant (TaBCAT1-A^Q50*^ TaBCAT1-B^R366−^) and WT (cv. Kronos) plants. For comparison, we also performed VIGS on *TaASIL2* in both plant backgrounds. We inoculated the plants with BSMV expressing either a 180-bp fragment of *TaASIL1* or a 300-bp fragment of *TaASIL2* that targeted conserved regions among the 2 *TaASIL1* or *TaASIL2* homoeologs (Figure S4a-b). Silencing efficiency was confirmed by RT-qPCR at 7 to 10 dpvi, with a significant reduction in *TaASIL1* or *TaASIL2* expression in the respective silenced plants compared to the negative controls (Figure S4c-d). RT-qPCR revealed that *TaASIL1* silencing in the *TaBCAT1* double mutant led to significantly higher *TaSIZ1* expression (log_2_ fold change of 1.40) and significantly lower *PR1* and *PR3* expression (log_2_ fold changes of −4.80 and −4.96) compared to the control. By contrast, no significant change in *TaSIZ1* or *PR1* expression was detected in WT plants subjected to *TaASIL1* silencing, although *PR3* displayed a minor increase (log_2_ fold change of 0.73) compared to the control (Fig. 6a).

Silencing *TaASIL2* in WT plants led to a significant increase (log_2_ fold change of 1.29) in *TaSIZ1* expression and significant decreases in *PR1* and *PR3* expression (log_2_ fold changes of −3.67 and −3.67, respectively). By contrast, silencing of *TaASIL2* in the *TaBCAT1* double mutant led to significant decreases in *TaSIZ1*, *PR1*, and *PR3* expression (log_2_ fold changes of −1.80, −5.10, and −6.32, respectively) compared to the controls (Figure S4e-g). Overall, the upregulation of *TaSIZ1* in the *TaBCAT1* mutant specifically following *TaASIL1* silencing suggests that TaASIL1 function is required to suppress *TaSIZ1* expression in this mutant. The further reduction in *TaSIZ1* and the decline in *PR1* and *PR3* expression in the *TaBCAT1* mutant following *TaASIL2* silencing suggest an additional unknown function for TaASIL2.

To further investigate the link between *TaSIZ1* and *TaASIL1* and/or *TaASIL2* expression, we conducted VOX of *TaASIL1* or *TaASIL2* in the WT and *TaBCAT1* double mutant (TaBCAT1-A^Q50*^ TaBCAT1-B^R366−^). We synthesised the full-length coding sequence of *TaASIL1-B* (1,251 bp) and *TaASIL2-B* (960 bp), which were independently cloned into the pCassRZ-BSMVy-yb2A-Lic vector (Kanyuka 2022) and performed BSMV-mediated VOX, using *BSMV::msc::iLov* as a positive control (Lee et al. 2015). Successful *TaASIL1* or *TaASIL2* overexpression was confirmed by RT-qPCR at 8 to 10 dpvi (average log_2_ fold change of 2.29 and 2.99, respectively; Figure S5a-b). RT-qPCR revealed that *TaASIL1* VOX in the *TaBCAT1* double mutant led to a significant reduction in *TaSIZ1* expression (log_2_ fold change of −1.41), whereas WT plants displayed a significant increase in *TaSIZ1* expression (log_2_ fold change of 1.24) compared to the control (Fig. 6b). VOX of *TaASIL2* in both the *TaBCAT1* double mutant and WT plants led to no significant change in *TaSIZ1* expression (Figure S5c). Since *TaSIZ1* expression was only repressed after *TaASIL1* overexpression in the *TaBCAT1* double mutant background, this suggests that *TaASIL1*-mediated *TaSIZ1* suppression may depend on the elevated BCAAs found in this mutant.

### Disrupting *TaASIL1-B* prevents Leu-triggered suppression of *TaSIZ1* expression

To explore the link between BCAAs, TaASIL1 and/or TaASIL2 function and the regulation of *TaSIZ1* expression, we identified Kronos TILLING mutants for *TaASIL1* and *TaASIL2* for comparison (Krasileva et al. 2017). We selected TILLING mutants in the B genome due to the 3-fold greater expression of the B homoeolog of *TaASIL1* (average 4.20 transcripts per million [tpm], S.D. 2.26) in 1,016 publicly available datasets compared to the A homoeolog (average 1.40 tpm, S.D. 1.09) (Borrill et al. 2016). For *TaASIL1-B*, we selected a B genome mutant (Kronos4594) harboring an early stop codon mutation at amino acid 31, and for *TaASIL2-B*, we selected a B genome mutant (Kronos 2311) with a missense variant at amino acid 191 that converts Gly to Ala (Figure S6a-b). We treated 14-day-old homozygous TaASIL1-B^W31*^ and TaASIL2-B^G191A^ plants with Leu (400 mg/L) and harvested them 24 h post-treatment, alongside mock-treated controls.

Following Leu treatment, TaASIL1-B^W31*^ plants displayed no significant change in *TaSIZ1* or *PR1* expression and a minor change in *PR3* expression (log_2_ fold change of 0.52) (Fig. 6c-e) and SA levels remained unchanged (Fig. 6f). Leu treatment of TaASIL2-B^G191A^ plants led to a minor increase in *TaSIZ1* expression and decline in *PR1* and *PR3* expression (log_2_ fold changes of 0.77, −2.02, and −0.87, respectively; Figure S6c-e). By contrast, Leu treatment of WT plants led to significantly suppressed *TaSIZ1* expression (>4 log_2_ fold) and significant upregulation of *PR1* and *PR3* (>6 and >2 log_2_ fold, respectively) (Fig. 6c-e and Figure S6c-e). Furthermore, we examined *TaASIL1* and *TaASIL2* expression in response to Leu treatment in WT plants. We found the expression of *TaASIL1*, and not *TaASIL2*, was significantly induced following Leu treatment (log_2_ fold change of 0.63) (Figure S6f). Overall, the lack of significant change in *TaSIZ1* expression following Leu treatment in the *TaASIL1* mutant and *TaASIL1* induction in WT plants in response to Leu treatment supports a role for TaASIL1 in regulating *TaSIZ1* expression in response to Leu.

### *TaASIL2* disruption enhances BCAA accumulation, *TaASIL1* expression, and *Pst* resistance

**Figure 7:**
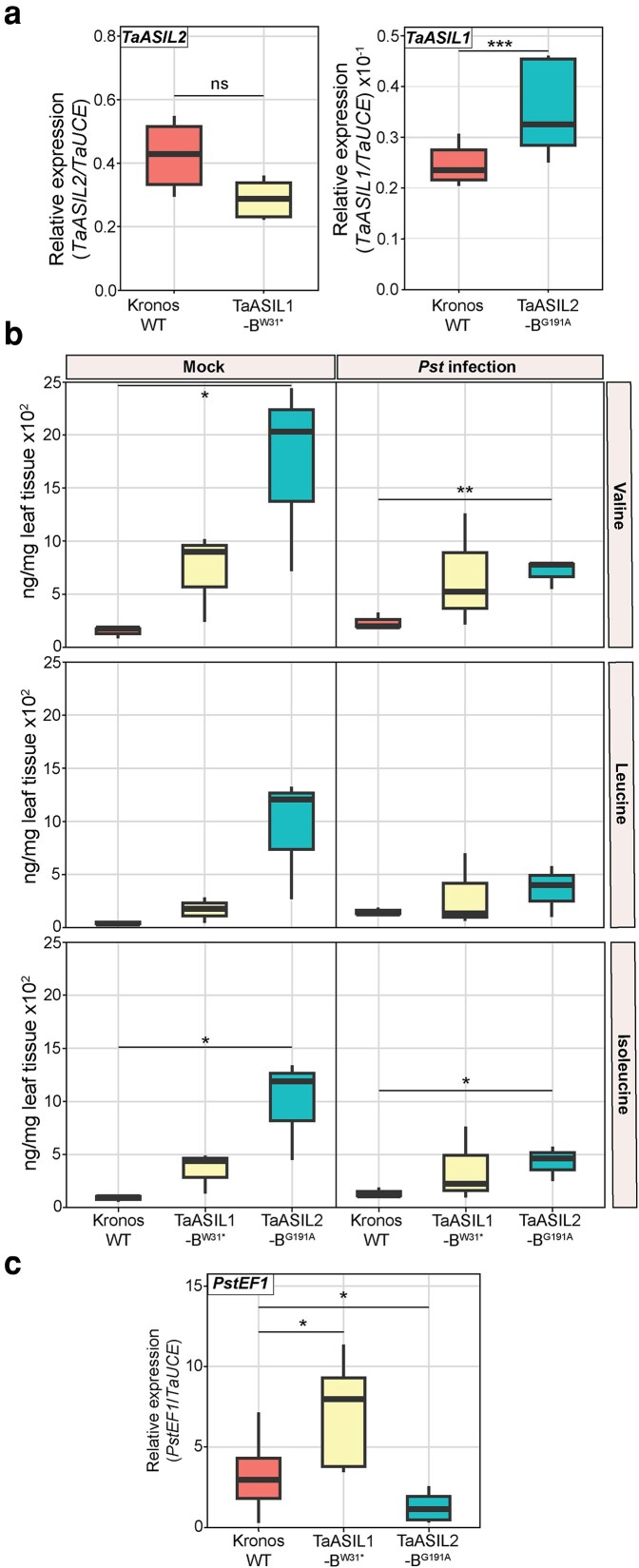
*TaASIL2* disruption leads to enhanced *TaASIL1* expression, BCAA accumulation, and a reduction in *Pst* biomass. a) RT-qPCR revealed *TaASIL1* expression was significantly elevated in TaASIL2-B^G191A^ plants (*n* = 3), with no change in *TaASIL2* expression found in TaASIL1-B^W31*^ plants (*n* = 3), when compared to WT plants (cv. Kronos, *n* = 3). b) TaASIL2-B^G191A^ plants (*n* = 3) in the presence and absence of *Pst* infection consistently showed a significant increase in BCAA levels (Val, Leu, Ile), when compared to the WT control (*n* = 3). No significant change was detected for TaASIL1-B^W31*^ plants (*n* = 3). *Pst-*infected plant samples were assessed 4 d post-inoculation (dpi). c) TaASIL2-B^G191A^ plants (*n* = 3) displayed a significant reduction in *Pst* biomass, and TaASIL1-B^W31*^ plants (*n* = 3) a significant increase in fungal biomass, when compared to the WT control (*n* = 3). RT-qPCR analysis was used to quantify *Pst* fungal biomass at 4 dpi with *Pst* by assessing the relative expression of the *PstEF1* gene, normalized to the *TaUCE* reference gene. Asterisks denote statistically significant differences between each pair of conditions (***: *P* < 0.001, **: *P* < 0.01; *: *P* < 0.05; 2-tailed *t*-test). Bar represents median value, box signifies the upper (Q3) and lower (Q1) quartiles, and whiskers are located at 1.5 times the interquartile range.

To investigate any potential interplay between the two TaASIL TFs that could explain the additional unknown function for TaASIL2 in *TaSIZ1* regulation, we examined *TaASIL1* and *TaASIL2* expression in the opposing TILLING disruption mutants. RT-qPCR analysis found no change in *TaASIL2* expression in TaASIL1-B^W31*^ plants. However, *TaASIL1* expression was significantly elevated in TaASIL2-B^G191A^ plants (log_2_ fold change of 1.12), indicating that disruption of *TaASIL2* may influence *TaASIL1* expression (Fig. 7a). As *TaASIL2* disruption can lead to elevated *TaASIL1* expression, this may explain the observed reduction in *TaSIZ1* expression in the *TaBCAT1* mutant following *TaASIL2* silencing. However, as *TaASIL1*-mediated repression of *TaSIZ1* expression was shown to require elevated BCAAs, we also measured the levels of the 3 BCAAs (Val, Leu, Ile) in the *TaASIL1* and *TaASIL2* disruption mutants. The levels of all 3 BCAAs were slightly elevated in both TaASIL1-B^W31*^ and TaASIL2-B^G191A^ plants (Fig. 7b). However, the only significant increases were detected in TaASIL2-B^G191A^ plants, with a log_2_ fold change of 3.39, 4.06, and 3.31 for Val, Leu, and Ile respectively.

To determine if the elevation in BCAAs levels, *TaASIL1* expression, and suppression of *TaSIZ1* in TaASIL2-B^G191A^ plants could impact *Pst* disease progression, we conducted seedling infection assays with TaASIL2-B^G191A^ plants and included TaASIL1-B^W31*^ plants for comparison, alongside a WT control (cv. Kronos). At 4 dpi, we employed RT-qPCR analysis to quantify relative *Pst* fungal biomass and found a significant increase in *Pst* biomass in TaASIL1-B^W31*^ plants when compared to the WT control (log_2_ fold change of 0.99) (Fig. 7c). In contrast, in comparison to WT, TaASIL2-B^G191A^ plants displayed a significant reduction in *Pst* biomass (log_2_ fold change of −1.81). Furthermore, we evaluated BCAA levels following *Pst* infection of TaASIL1-B^W31*^, TaASIL2-B^G191A,^ and WT plants. In the WT control plants, *Pst* infection led to a slight elevation in BCAA accumulation, with a significant increase in Leu levels (log_2_ fold change of 2.06). However, in comparison to *Pst* infected WT plants, *Pst* infection led to similar results seen in uninfected plants with a slight elevation of BCAA levels in both TaASIL1-B^W31*^ and TaASIL2-B^G191A^ plants, with significant increases only detected in TaASIL2-B^G191A^ plants, with a log_2_ fold change of 1.52, 0.92 and 1.55 for Val, Leu and Ile respectively (Fig. 7b). These results indicate that the disruption of *TaASIL2* increases BCAAs accumulation, *TaASIL1* expression and enhances *Pst* resistance, likely due to induction of TaASIL1-mediated suppression of *TaSIZ1* expression.

## Discussion

BCAAs play crucial roles in plant defense, serving as precursors for many secondary metabolites involved in plant immunity (Rizhsky et al. 2002). Fluctuations in BCAA levels are also early indicators of pathogen attack, triggering an immune response (Moormann et al. 2022). However, how BCAA fluctuations initiate immune signaling has remained unclear. In wheat, elevated BCAA levels resulting from *TaBCAT1* disruption were previously linked to increased SA levels, upregulated *PR* gene expression, and a reduction in *Pst* and *Pgt* infection. *TaBCAT1* encodes a BCAA aminotransferase thought to be involved in the catabolism of BCAAs to acetyl-CoA in mitochondria (Corredor-Moreno et al. 2021). Here, we demonstrate that the wheat ortholog of the SUMO E3 ligase SIZ1 (TaSIZ1), which acts as an SA regulator, is suppressed in *TaBCAT1* disruption mutants, likely as a response to BCAA accumulation. In addition, we found that treatment of WT plants with the BCAA Leu could similarly repress *TaSIZ1* expression. Thus, illustrating that BCAAs—which accumulate during pathogen attack—can act to elevate SA-mediated defence responses through suppression of TaSIZ1 activity. SIZ1 is an essential component of the SUMOylation system and directly promotes the reversible covalent binding of SUMO molecules to the Lys residue(s) of substrate proteins to regulate their activity, localization, stability, and/or protein–protein interactions (Fang et al. 2022). SIZ1 activity directly contributes to plant growth, development, abiotic stress responses, and SA-mediated innate immunity (Sharma et al. 2021). Thus, we propose that the increased SA levels in the *TaBCAT1* disruption mutants and their enhanced resistance to *Pst* and *Pgt* result from BCAA-mediated suppression of *TaSIZ1* expression, and the resulting disruption of SA-mediated immunity usually mediated by TaSIZ1 activity (Lee et al. 2007).

**Figure 8:**
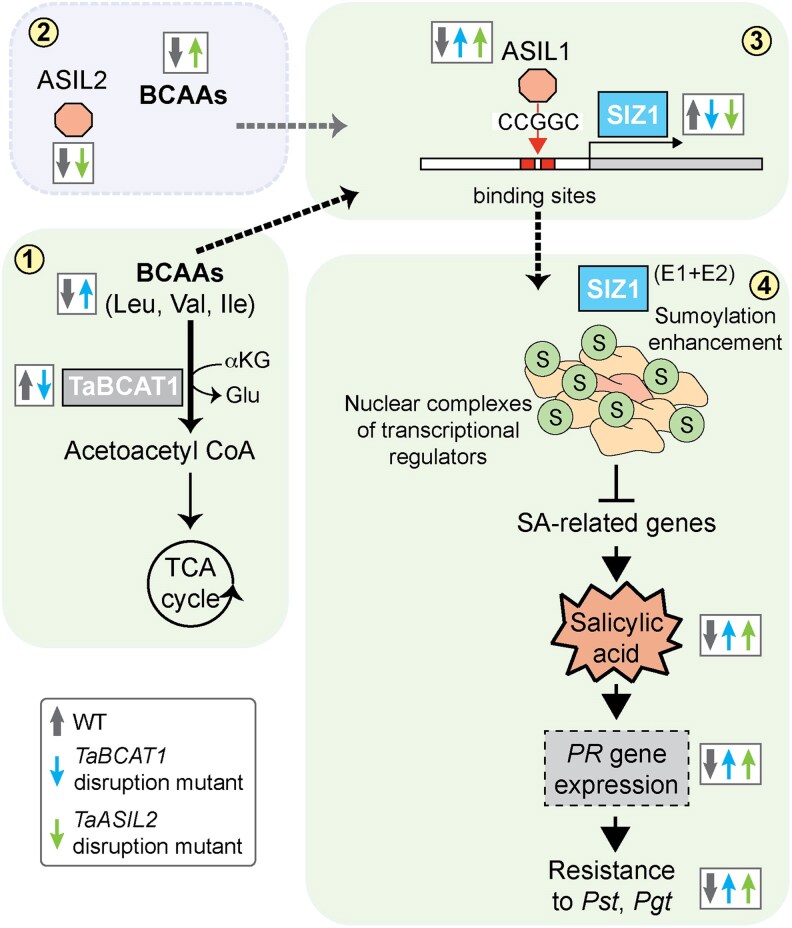
Model illustrating the role of TaSIZ1 in linking elevated BCAA levels to SA accumulation. Disrupting *TaBCAT1* (blue arrow) increases BCAA levels (1), that are also elevated in response to disruption of the TF TaASIL2 (green arrow) (2). In response, the TF TaASIL1 represses *TaSIZ1* expression (3). This, in turn, enhances free salicylic acid (SA) accumulation and pathogenicity-related (*PR*) gene expression, thereby increasing resistance to *Pst* and *Pgt* infection (4) (Corredor-Moreno et al. 2021). The grey arrow represents the wild-type (WT). BCAAs, branched-chain amino acids; TCA, tricarboxylic acid; 5′-CCGGC-3′, predicted TaASIL1 binding site in the *TaSIZ1* promoter.

In Arabidopsis, AtSIZ1 regulates SA-mediated defense signaling, and its loss of function results in increased SA levels, heightened *PR* gene expression, and enhanced EDS1/PAD4-dependent bacterial disease resistance (Lee et al. 2007). Similarly, silencing *TaSIZ1* in WT plants resulted in elevated SA levels and a reduction in *Pst* infection, supporting an analogous role for TaSIZ1 as a negative regulator of SA-mediated defense signaling. Overexpressing *TaSIZ1* in *TaBCAT1* disruption mutants reduced the typically heightened SA levels, supporting the notion that *TaSIZ1* suppression in these mutants is critical for SA accumulation (Fig. 8). SIZ1 activity and the subsequent transcriptional modification of related substrates increase in response to various environmental cues, including cold stress (Miura et al. 2007), phosphate starvation (Miura et al. 2005), and high copper levels (Chen et al. 2011), among others. Conversely, *TaSIZ1* expression in WT plants was repressed in response to treatment with the BCAA Leu; exogenous application of 400 mg/L Leu led to a log_2_ fold reduction of −1.02 in *TaSIZ1* expression in WT plants, which subsequently increased *PR* gene expression and SA levels. The buildup of BCAAs, such as Leu, in response to a reduction in BCAT expression and/or function is increasingly recognized as playing an important—though previously unclear—role in initiating SA-mediated defense responses during both abiotic (Buffagni et al. 2020) and biotic stress (Corredor-Moreno et al. 2021). Our data suggest that this critical link between BCAA accumulation and the activation of SA signaling could be mediated by suppressed *TaSIZ1* expression.

SUMOylation is a well-established regulator of transcription. SIZ1 was shown to influence the SUMOylation of over 100 proteins, including various chromatin modifiers linked to abiotic and biotic stress responses (Rytz et al. 2018). However, few TFs that regulate SUMOylation machinery components, such as SIZ1, in response to environmental cues in plants have been identified. One study in apple (*Malus domestica Borkh*) identified a low-temperature-responsive MYB TF (MdMYB2) that interacts directly with the *MdSIZ1* promoter to activate its expression (Jiang et al. 2022). The MYB TF family, one of the largest TF families in plants, is highly functionally diverse and is characterized by the presence of a conserved MYB DNA-binding domain (Ambawat et al. 2013). In the current study, comparative nuclear proteomic analysis of a *TaBCAT1* mutant identified two potential MYB/SANT TFs that were enriched and corresponded to DNA-binding motifs in the *TaSIZ1* promoter sequence. These two enriched TFs were predicted to be orthologous to the trihelix TFs ASIL1/2. In Arabidopsis, ASIL1 and ASIL2 were originally linked to the repression of embryo maturation during vegetative growth (Gao et al. 2009). However, this association has since been refuted, and they are now thought to function in abiotic and biotic stress responses (Ruiz et al. 2021). Accordingly, TaASIL1 directly bound to cis-acting elements in the *TaSIZ1* (and *AtSIZ1*) promoter, pointing to a potential role for TaASIL1 in regulating SA-mediated defense responses by modulating *TaSIZ1* expression. Furthermore, we found that TaASIL2 function is required to maintain BCAA homeostasis, with Val, Leu, and Ile hyperaccumulating in *TaASIL2* disruption mutants, which also displayed elevated *TaASIL1* expression and reduced *Pst* infection. Thus, although TaASIL2 appears unable to directly regulate *TaSIZ1* transcription, we propose that TaASIL2 may also indirectly regulate TaASIL1-mediated suppression of *TaSIZ1* expression (Fig. 8). This suggests that MYB TFs may play key roles in transducing a diverse array of environmental signals to modulate SIZ1 activity in plants.

Although numerous cues are known to activate TFs, the effect of fluctuating amino acid levels on TF activity remains poorly understood. For example, Gln treatment upregulated several TF genes in rice (including MYB-like TFs), suggesting that Gln functions as a signaling molecule that regulates plant growth and stress responses (Kan et al. 2015). Here, we showed that Leu treatment can induce *TaASIL1* expression and that repression of *TaSIZ1* expression during Leu treatment is impaired in *TaASIL1* disruption mutants, with only minor reductions in *PR* gene expression and no change in associated SA levels. In addition, overexpression of *TaASIL1* only led to suppression of *TaSIZ1* expression in the *TaBCAT1* mutant, further indicating that TaASIL1-mediated *TaSIZ1* suppression is likely dependent on BCAA availability. We also demonstrated that disruption of *TaASIL2* can lead directly to BCAA accumulation, TaASIL1-mediated *TaSIZ1* suppression, and enhanced *Pst* resistance. This suggests that the trihelix MYB/SANT TF TaASIL1 plays a critical role in linking BCAA accumulation to SA-mediated defense responses via *TaSIZ1* repression in wheat. Overall, this study highlights how BCAA levels mediate the transcriptional regulation of *TaSIZ1* via TaASIL1 activity (Fig. 8) and provides important insight into how BCAA accumulation leads to constitutive SAR activation and enhanced *Pst* and *Pgt* resistance following *TaBCAT1* disruption. These findings provide deeper insight into the mechanism underlying the resistance conferred by disrupting *TaBCAT1*, which is a promising target for enhancing wheat resilience to rust infection.

## Methods

### Identification and annotation of the wheat ortholog of *SIZ1*

The wheat (*T. aestivum*) *SIZ1* ortholog (termed *TaSIZ1*) was identified in EnsemblPlants (Bolser et al. 2016) using the search term “SUMO E3 ligase” and further confirmed through sequence similarity searches using the *Arabidopsis thaliana* homolog (*AtSIZ1*; AT5G60410) as a query in a BLASTp search of the wheat reference genome (RefSeq v2.1) (Zhu et al. 2021). *SIZ1* orthologs in various species were identified using EnsemblPlants (Bolser et al. 2016) with a sequence identity cutoff of >50%. Phylogenetic analysis was conducted using FastTree version 2.1 (Price et al. 2010) with an approximate maximum likelihood approach, and trees were visualized using iTOL version 7 (Letunic and Bork 2024). The SAP, SP-RING, and PHD domains of TaSIZ1 and AtSIZ1 were identified and annotated using NCBI's Entrez database (Sayers et al. 2025). Each domain was aligned and scored using MAFFT (Multiple Alignment using Fast Fourier Transform) software version 6.717b in Biopython (Katoh et al. 2002). The PINIT and S-x-S domains were manually annotated.

### Virus-mediated modification of gene expression using the BSMV

For VIGS, a 216-bp fragment of *TaSIZ1*, a 180-bp fragment of *TaASIL1*, and a 300-bp fragment of *TaASIL2* were selected to target the 2 homoeologous copies of each gene simultaneously using siRNA-Finder software (Luck et al. 2019). For VOX, the full-length 2,538-bp CDS of *TaSIZ1* (TraesCS1A02G065700.1), 1,251-bp CDS of *TaASIL1* (TraesCS3B02G305100), or 960-bp CDS of *TaASIL2* (TraesCS6B02G230000) was selected. Each fragment was synthesized and cloned into the BSMV vector pCa-γbLIC for VIGS (Yuan et al. 2011) or pCassRZ-BSMVy-yb2A-LIC for VOX (Kanyuka 2022) by Genewiz (Azenta Life Sciences, MA, USA). *BSMV::TaPDS* was used as a positive control and *BSMV::msc4D* or *BSMV::msc::iLov* as a viral infection control for VIGS and VOX, respectively (Lee et al. 2015). All constructs were independently transformed into *Agrobacterium tumefaciens* strain GV3101 using electroporation and transiently expressed in *Nicotiana benthamiana* leaves as described previously (Bos et al. 2006). Sap was harvested from *N. benthamiana* leaves once BSMV-induced viral mosaic symptoms were visible, approximately 7 to 8 dpvi. Approximately 14-day-old wheat seedlings (cv. Kronos) grown in a glasshouse under long-day conditions (16-h-light/8-h-dark photoperiod and 19 °C/14 °C cycles) were inoculated with the harvested sap using mechanical abrasion (Lee et al. 2015) and transferred to a controlled environment cabinet (MLR-352-PE, PHCbi, Panasonic Corporation, Kadoma, Japan) with a 16-h-light/8-h-dark photoperiod and 23 °C/16 °C cycles with LED fluorescent tubes (Fusion Lamps, Doncaster, UK). Wheat leaf samples were collected to evaluate gene expression 7 to 10 dpvi using RT-qPCR with gene-specific primers targeting regions outside of the target region used for VIGS (Table S2).

### RT-qPCR

RNA was extracted from ∼100 mg of fresh wheat leaf tissue using a Qiagen Plant RNeasy Mini Kit (Qiagen, Manchester, UK) according to the manufacturer's instructions. The integrity of the RNA was assessed using a Qubit fluorometer (Thermo Fisher Scientific, Paisley, UK), and prior to cDNA synthesis, <10 µg/µL of RNA per sample was treated with 1 U/μL DNase I in a 50-mL reaction volume (Thermo Fisher Scientific, Paisley, UK). First-strand cDNA was synthesized using a Verso cDNA Synthesis Kit (Thermo Fisher Scientific, Paisley, UK) using oligoDT primers. qPCR was performed using Light Cycler 480 SYBR Green I Master Mix (Roche, Basel, Switzerland) following the manufacturer's instructions using primers at a final concentration of 0.5 µM. Data acquisition was performed using Light Cycler 480 SW 1.5 software (Roche, Basel, Switzerland), and relative transcript levels were determined by comparing the ΔCt values between the target gene and wheat *TaUCE* (for ubiquitin-conjugating enzyme) (Borrill et al. 2016). Each experiment was performed using 3 to 4 biological replicates (separate plants), with 2 to 3 technical replicates per plant. Primers used for RT-qPCR are listed in Table S2.

### Quantification of free SA

Total free SA content was measured in 100 mg of fresh leaf tissue from 14-day-old wheat seedlings as described previously (Corredor-Moreno et al. 2021) using ultra-performance liquid chromatography–mass spectrometry (UPLC/MS). Data acquisition and analysis were performed using TargetLynx (Waters, Wilmslow, UK).

### *Pst* seedling infection assays

Seedlings subjected to VIGS for *TaSIZ1* were inoculated with *Pst* urediniospores (isolate F22; Hubbard et al. 2015)) at 14 dpvi, with 3 biological replicates performed (separate plants). Approximately 10 mg of fresh *Pst* urediniospores were heat-activated at 40 °C for 5 min and resuspended in Novec 7100 (Sigma-Aldrich, USA) to 1 mg/mL and used for spray inoculation. Following treatment, seedlings were kept in the dark at 10 °C, with near 100% relative humidity for 24 h before being returned to glasshouse growth conditions (16-h-light/8-h-dark photoperiod and 19 °C/14 °C cycles). Leaf samples were harvested 4 dpi with *Pst,* and fungal biomass was assessed using RT-qPCR as described above. *Pst* infection assays were also conducted on TaASIL1-B^W31*^ and TaASIL2-B^G191A^ plants. Seeds of the mutant lines and the WT Kronos variety were pre-germinated on pre-wetted filter paper, sown in 9-cm pots, and grown in a glasshouse under long-day conditions (16-h light/8-h dark photoperiod and 19 °C/14 °C cycles). Seedlings were inoculated with *Pst* urediniospores (isolate F22) as described above, with 3 biological replicates performed (separate plants). For control experiments, a mock spray inoculation with Novec 7100 alone was performed. RT-qPCR was performed to assess fungal biomass on leaves harvested at 4 dpi as described above.

### Exogenous application of BCAAs

Fourteen-day-old WT wheat seedlings (cv. Kronos) grown in a glasshouse (16-h-light/8-h-dark photoperiod and 19 °C/14 °C cycles) were treated with Val, Leu, or Ile individually across a series of concentrations (10, 50, 100, 200, and 400 mg/L) via foliar spraying or mock-treated with water. Plants were transferred to a controlled environment cabinet (MLR-352-PE, PHCbi, Panasonic Corporation, Kadoma, Japan) with a 16-h-light/8-h-dark photoperiod and 23 °C/16 °C cycles with LED fluorescent tubes (Fusion Lamps, Doncaster, UK). Leaves were collected from each experimental group 12, 24, and 48 h post-treatment and analyzed by RT-qPCR for *TaSIZ1*, *PR1*, and *PR3* expression, and a subset of samples was examined by UPLC/MS to assess SA levels. In addition, WT (cv. Kronos), TaASIL1-B^W31*^, and *TaASIL2-B^G191A^* plants were treated with Leu (400 mg/L) as described above, harvested 24 h post-treatment, and *TaSIZ1*, *PR1*, and *PR3* expression and SA levels examined. Each experimental group consisted of 3 to 4 biological replicates (separate plants) with 3 technical replicates.

### Nuclear proteomics of the *TaBCAT1* disruption mutant

Leaves of four 14-day-old seedlings of the *TaBCAT1* double mutant (TaBCAT1-A^Q50*^ TaBCAT1-B^R366−^) and 4 WT plants (cv. Kronos) grown in a glasshouse under long-day conditions (16-h-light/8-h-dark photoperiod and 19 °C/14 °C cycles) were harvested and frozen in liquid nitrogen. For each sample, 1 mL of ice-cold nuclei isolation buffer (100 mM Trizma, 800 mM KCl, 100 mM EDTA, 17 mM spermine, 17 mM spermidine, 500 µM sucrose, 320 μM Triton X-100, and 2.78 μM PVP360) was added to 100 mg of powdered leaf tissue. The samples were mixed thoroughly for 15 min at 150 rpm in a thermomixer (Eppendorf, Hamburg, Germany), and the lysate was filtered using a Steriflip^®^ filter unit with a 20-µm pore size (Merck Millipore, Watford, UK). The filtered lysates were centrifuged (7,000*×g*) for 20 min at 4 °C. After adding 100 µL of ice-cold homogenization buffer (containing 100 mM Trizma, 800 mM KCl, 100 mM EDTA, 17 mM spermine, 17 mM spermidine, and 500 µM sucrose) to each pellet, the samples were homogenized and centrifuged (7,000*×g*) for 5 min at room temperature and the supernatant discarded. Protein pellets were prepared for nanoLC-MS/MS as described previously (Mateo-Bonmatí et al. 2024) using an Orbitrap Eclipse^TM^ Tribrid^TM^ mass spectrometer coupled to an UltiMate 3000 RSLCnano LC system (Thermo Fisher Scientific, Hemel Hempstead, UK). Raw MS data were processed and quantified with Proteome Discoverer 3.1 (Thermo Fisher Scientific, Hemel Hempstead, UK) using the search engine CHIMERYS (MSAID, Munich, Germany) as described (Mateo-Bonmatí et al. 2024), using the UniProt *T. aestivum* filtered proteome (UP000019116; 130,673 entries) (Bateman et al. 2019). For quantification, 4 replicates per condition were measured (Mateo-Bonmatí et al. 2024), and ratios were calculated using the 3 most abundant peptides per sample, with significance assessed using a *t*-test; those with an adjusted *P*-value < 0.05 were considered significant. Results were exported into Microsoft Excel, including data for protein abundances, ratios, *P*-values, number of peptides, protein coverage, the CHIMERYS identification score, and other values for further analyses. Ratios of abundance between the *TaBCAT1* double mutant (TaBCAT1-A^Q50*^ TaBCAT1-B^R366−^) and WT (cv. Kronos) samples were extracted and used to categorize proteins with higher abundance in the *TaBCAT1* double mutant (<1) and those with higher abundance in the WT (>1). Each protein set was subjected to GO enrichment analysis using the GOATOOLS Python package (Klopfenstein et al. 2018).

### Annotation of DNA-binding motifs in the *TaSIZ1* promoter

A 2-kb sequence upstream of the *TaSIZ1* 5′UTR was retrieved from the Grain Genes database (Yao et al. 2022), and MEME suite version 5.5.7 (Bailey et al. 2015) was used to identify conserved sequence motifs using the Arabidopsis database and the parameters of optimum motif width of ≥6 and ≤50 and maximum number of motifs of 20. For each identified motif, the predicted corresponding TFs from Arabidopsis were searched against the Chinese Spring wheat reference genome (RefSeq v2.1) (Zhu et al. 2021) using BLASTp to identify wheat orthologs. The UniProt IDs, GO terms, and sequences were retrieved from UniProt as FASTA files (Bateman et al. 2019) for each wheat ortholog. TF families were identified using PlantTFDB version 5.0 (Jin et al. 2017) and by searching the Interpro database (Paysan-Lafosse et al. 2023). To predict TF binding motifs in the *TaSIZ1* promoter, a 2-kb sequence upstream of the *TaSIZ1* 5′UTR was analyzed using the PlantCare database (Lescot et al. 2002). A potential binding motif corresponding to TaASIL1 and TaASIL2 (trihelix members of the MYB/SANT family) was further examined in the *TaSIZ1* and *AtSIZ1* promoter sequences using the PlantPAN database version 4.0 (Chow et al. 2024).

### Identification and annotation of TaASIL1 and TaASIL2

Gene sequences were extracted from the wheat reference genome (RefSeq v2.1) (Zhu et al. 2021) for 2 proteins (UniProt IDs A0A077RZA1 and A0A3B6PN54) enriched in the nuclear proteomic data of the *TaBCAT1* double mutant and with binding sites in the *TaSIZ1* promoter (TraesCS3B02G305100 and TraesCS6B02G230000). Sequence conservation with Arabidopsis MYB/SANT TFs AtASIL1 (AT1G54060) and AtASIL2 (AT3G14180) was confirmed through a BLASTp search against the Arabidopsis Information Resource database (Berardini et al. 2015) using TraesCS3B02G305100 and TraesCS6B02G230000 as queries. Phylogenetic analysis was conducted on 80 previously defined wheat trihelix TFs (including TaASIL1 and TaASIL2), AtASIL1 and AtASIL2 from Arabidopsis, and NtSIP1 from tobacco (*Nicotiana tabacum*) using FastTree version 2.1 (Price et al. 2010) and an approximate maximum likelihood approach. Trees were visualized using iTOL version 7 (Letunic and Bork 2024). The trihelix and α-helix domains of TaASIL1, TaASIL2, AtASIL1, AtASIL2, and NtSIP1 were annotated using NCBI's Entrez database (Sayers et al. 2025).

### Yeast one-hybrid assay

The full-length CDSs of *TaASIL1* (1,879 bp) and *TaASIL2* (2,375 bp) were synthesized, and Gateway cloning–compatible entry clones were generated in the pDONR221 vector (BioCat GmbH, Heidelberg, Germany). *TaASIL1* and *TaASIL2* were transferred from the entry clones into the yeast expression vector pDEST-GADT7 (Arabidopsis Biological Resource Center, Ohio State University, Ohio, USA) by LR cloning (Invitrogen, Paisley, UK). For *TaSIZ1* and *AtSIZ1*, a 2-kb fragment immediately upstream of the 5′UTR was amplified from wheat DNA (cv. Kronos) or Arabidopsis DNA (accession Columbia-0) and cloned independently in fusion with the minimal *HIS3* promoter in the pHIS2 vector (Gentaur, Potters Bar, UK) (Table S3). As a positive control, the 701-bp *TaNF-YB1* gene (TraesCS6B02G316700) was amplified from wheat cDNA (cv. Kronos) and introduced into the pDEST-GADT7 vector, and the 400-bp promoter of a wheat phytosulfokine gene (*TaPSK1-A*) was amplified and introduced into the pHIS2 vector (Table S3). The construct pairs were transformed into yeast (*Saccharomyces cerevisiae*) strain Y187 using the lithium acetate method (Gietz and Schiestl 2007). Yeast transformants were initially grown in synthetic defined (SD) yeast medium devoid of Leu and Trp and supplemented with His at 20 μg/mL (SD/−Leu/−Trp) to assess successful transformation. To measure interactions, transformants were grown on triple dropout selective medium (TDO: SD/−Trp/−Leu/−His) supplemented with 20 to 25 mM 3-AT (3-amino-1,2,4-triazole), a selective inhibitor of His biosynthesis. The yeast strains were grown at 28 °C and photographed after 7 to 10 d.

### Selection of *TaASIL1* and *TaASIL2* wheat TILLING mutant lines

Tetraploid wheat TILLING lines (cv. Kronos) (Krasileva et al. 2017) with disruptions in *TaASIL1* and *TaASIL2* in the B genome were selected. The Kronos4594 (termed TaASIL1-B^W31*^) and Kronos 2311 (termed TaASIL2-B^G191A^) mutant lines harboring an early stop codon mutation in TaASIL1 at amino acid 31 or a missense variant in TaASIL2 at amino acid 191 that converts glycine to alanine were selected. WT and mutant seeds were pregerminated on prewetted filter paper, sown in 9-cm pots, and grown in a glasshouse under long-day conditions (16-h-light/8-h-dark photoperiod and 19 °C/14 °C cycles). DNA was extracted from leaf tissue harvested from 14-day-old TILLING mutants and WT plants using a Qiagen Plant DNeasy Mini kit (Qiagen, Manchester, UK) according to the manufacturer's instructions. Genotyping was carried out using subgenome-specific primer sequences and Kompetitive allele-specific PCR (KASP, LGC Genomics, Teddington, UK) as described previously (Ramirez-Gonzalez et al. 2015) (Table S4).

### Quantification of soluble BCAA levels

Fourteen-day-old seedlings of the TaASIL1-B^W31*^ and TaASIL2-B^G191A^ mutants and Kronos WT were subjected to *Pst* infection as described above. At 4 dpi, tissue from all 3 lines were harvested alongside 14-day-old seedlings of the same lines without *Pst* infection. Samples were flash-frozen in liquid nitrogen, and an average of 15 mg of plant tissue per sample was lyophilized for 48 h. Soluble BCAAs were extracted and quantified from all samples as described previously (Corredor-Moreno et al. 2021). In brief, a 60 µL solution containing 20 mM HEPES (pH 7.0), 5 mM EDTA, and 10 mM NaF, and 250 µL of chloroform/methanol (1.5/3.5, v/v) were added to the lyophilized plant material. Samples were homogenized using the TissueLyser II (Qiagen, Manchester, UK) with one Tungsten 3 mm bead per tube and then incubated on ice for 30 min. The homogenate was centrifuged at 15,871 g for 10 min, and the aqueous phase was collected. The dried residue was washed twice with 300 µL water, centrifuged, and diluted 1 to 100 in water. A 10 µL aliquot was derivatized using Waters' AccQ tag kit according to the manufacturer's instructions (Waters, Wilmslow, UK), and 2 µL was used for injection. Separation of amino acids in the sample extract was on a 100 mm × 2.1 mm, 2.7 μm Kinetex XB-C18 column (Phenomenex, CA, USA) using a 14.5 min gradient of 1 to 20% acetonitrile versus 0.1% formic acid in water, run at 0.58 mL. min-1 and 25 °C. Mass transitions were as described previously (Corredor-Moreno et al. 2021). Each experiment was performed using 3 biological replicates (separate plants).

### Accession numbers

Sequence data from this article can be found in the GenBank/EMBL (Arabidopsis) or EnsemblPlants (wheat) libraries under the following accession numbers: *AtSIZ1*, AT5G60410; *TaSIZ1*-A, TraesCS1A02G065700; *TaSIZ1-B*, TraesCS1B02G083900; *AtASIL1*, AT1G54060; *AtASIL2*, AT3G14180; *TaASIL1*, TraesCS3B02G305100; *TaASIL2*, TraesCS6B02G230000.

## Supplementary Material

koag135_Supplementary_Data

## Data Availability

All custom computer code and the Newick files for the phylogenies have been deposited at GitHub (https://github.com/SaundersLab/SIZ1-paper). Additional data that support the findings of this study are available in the Supplementary material of this article (Supplemental Data Set S6).
