# Supplementary material for: A wheat MYB transcription factor activates defense by repressing TaSIZ1 in response to branched-chain amino acid accumulation
Source: Plant Cell. 2026 May 6;38(5):koag135. doi: 10.1093/plcell/koag135 (PMC13196585; doi:10.1093/plcell/koag135)
Supplement: koag135_Supplementary_Data [file koag135_supplementary_data.zip › Supplementary Data.pdf]

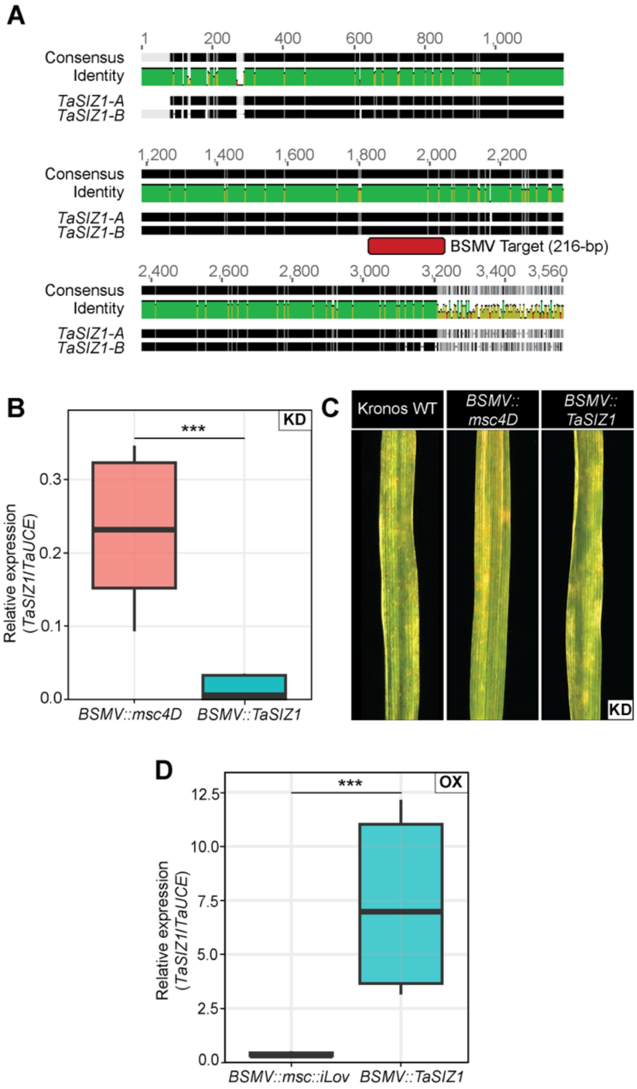

Supplemental Figure 1: Virus-induced gene silencing and overexpression of *TaSIZ1*. (Supports Figure 1.) **A.** A 216-bp fragment of *TaSIZ1* was designed to target the two homoeologous copies of the gene simultaneously in tetraploid wheat (cv. Kronos) for silencing. **B.** Virus-induced gene silencing (VIGS) of *TaSIZ1* significantly reduced *TaSIZ1* expression at 8 days post-viral inoculation (dpvi) in wild-type (cv. Kronos) plants. *TaSIZ1* expression levels were measured by RT-qPCR in plants silenced with *BSMV::TaSIZ1* and compared to the negative control; *BSMV::msc4D* was used as a viral infection control. Four samples each were evaluated for *BSMV::TaSIZ1* and *BSMV::msc4D.* **C.** *Pst* pustule development was inhibited following VIGS of *TaSIZ1*. Plants were inoculated with *BSMV::TaSIZ1* (n = 3) or *BSMV::msc4D* (n = 3), and at 14 dpvi plants were infected with *Pst,* alongside Kronos WT plants (n = 3)*.* Images were captured 14 days post inoculation with *Pst*. **D.** Virus-induced overexpression (VOX) of *TaSIZ1* significantly increased *TaSIZ1* expression 8–10 dpvi in *TaBCAT1* double disruption mutant (TaBCAT1-A^{Q50*} TaBCAT1-B^{R366-}) plants. *TaSIZ1* expression was assessed by RT-qPCR in plants subjected to VOX of *TaSIZ1* (n = 3) and compared to *BSMV::msc::iLov* (n = 3) as a positive fluorescent protein control (Lee et al., 2015). Asterisks denote statistically significant differences between each pair of conditions (***: p < 0.001; two-tailed t-test). KD, silencing; OX, overexpression. Bar represents median value, box signifies the upper (Q3) and lower (Q1) quartiles, and whiskers are located at 1.5 times the interquartile range.

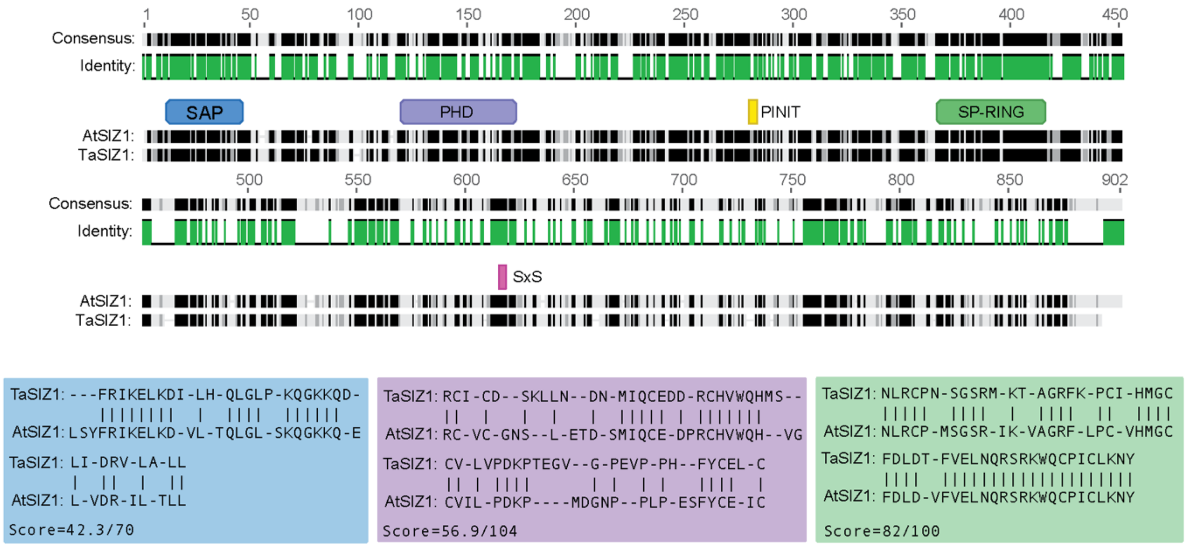

Supplemental Figure 2: Sequence alignment of TaSIZ1 and AtSIZ1. (Supports Figure 2.) Both TaSIZ1 and AtSIZ1 show a typical conserved modular architecture, with five structural elements: (i) a putative N-terminal Scaffold Attachment Factor A/B, Acinus and PIAS (SAP) DNA-binding domain, (ii) a zinc finger PHD (for plant homeodomain), (iii) a PINIT (for Pro-Ile-Asn-Ile-Thr) motif, (iv) a central SP-RING (Siz/PIAS-RING) domain, and (v) a C-terminal S-X-S motif. Full amino acid sequence alignments of the SAP (blue), PHD (purple), and SP-RING (green) domains are shown.

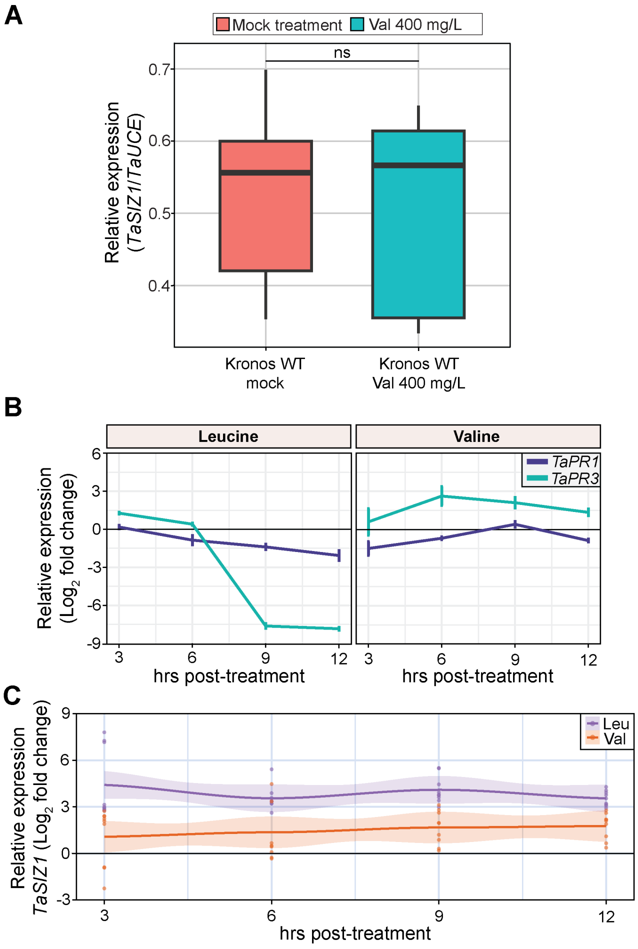

Supplemental Figure 3: Twenty-four hours post Val treatment no significant change in *TaSIZ1* was detected, with no change also found in *TaSIZ1, PR1 or PR3* expression at 3, 6, 9 and 12 hours following Leu or Val treatment. (Supports Figure 3.) **A.** RT-qPCR analysis indicated no change in *TaSIZ1* expression 24 hours post Val treatment (400 mg/L). In addition, no significant changes in *PR1*, *PR3* (**B**) or *TaSIZ1* (**C**) expression was found at 3, 6, 9 and 12 hours post Leu and Val treatment (400 mg/L). Mock, Val and Leu treatment n = 3. Box plots: bar represents median value, box signifies the upper (Q3) and lower (Q1) quartiles, and whiskers are located at 1.5 times the interquartile range.

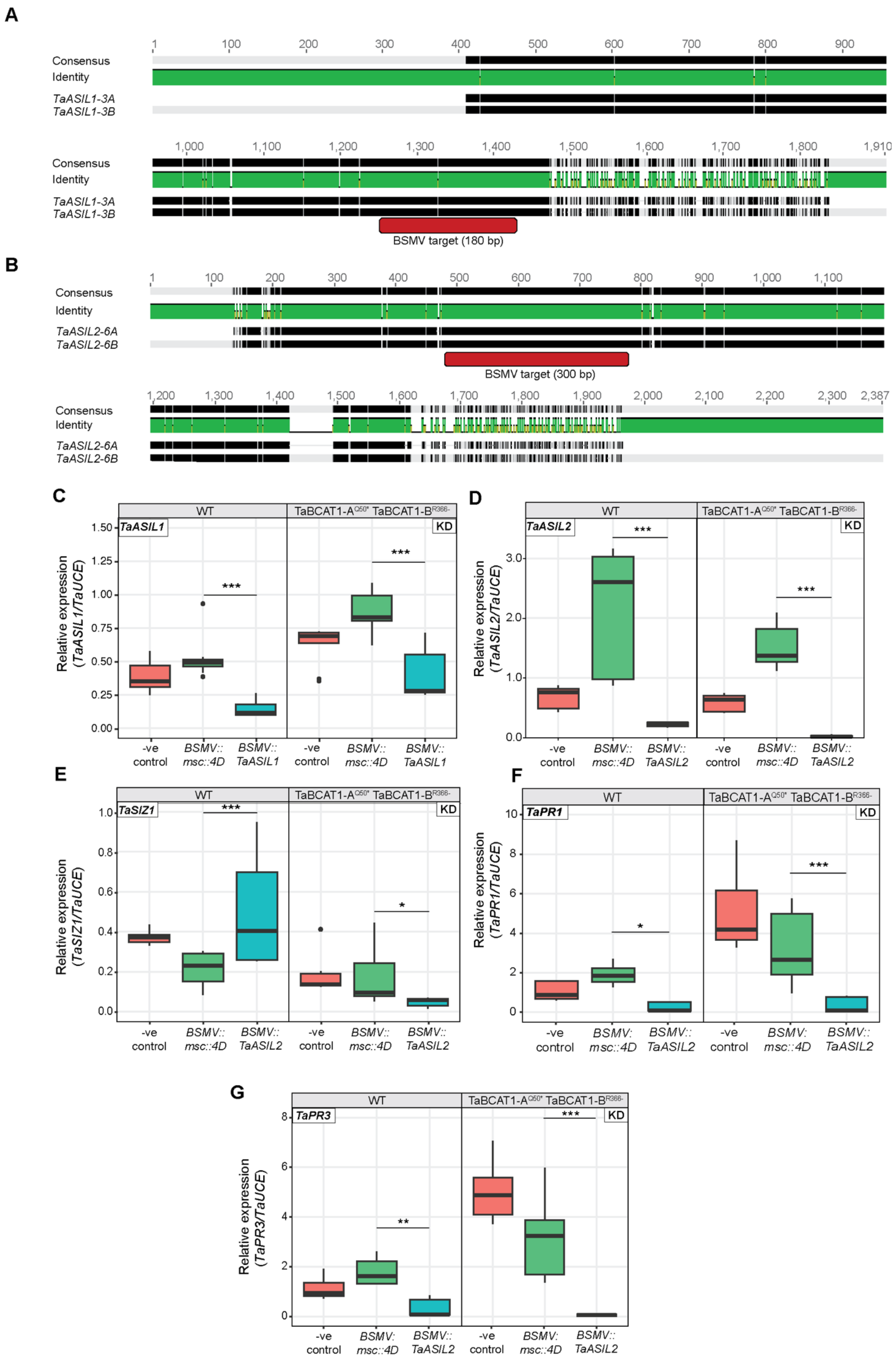
A
Consensus
Identity
TaASIL1-3A
TaASIL1-3B
BSMV target (180 bp)
B
Consensus
Identity
TaASIL2-6A
TaASIL2-6B
BSMV target (300 bp)
C
WT
KD
TaASIL1
Relative expression (TaASIL1/TaUCE)
-ve control
BSMV::msc::4D
BSMV::TaASIL1
D
TaASIL2
Relative expression (TaASIL2/TaUCE)
BSMV::TaASIL2
E
TaSIZ1
Relative expression (TaSIZ1/TaUCE)
F
TaPR1
Relative expression (TaPR1/TaUCE)
G
TaPR3
Relative expression (TaPR3/TaUCE)

Supplemental Figure 4: Virus-induced gene silencing (VIGS) of *TaASIL1* and *TaASIL2*. (Supports Figure 6.) **A-B.** The 180-bp (**A**) and 300-bp (**B**) fragments designed to target the two homoeologous copies of *TaASIL1* or *TASIL2* simultaneously in tetraploid wheat (cv. Kronos). **C-D.** *TaASIL1* and *TaASIL2* expression was significantly reduced 7–10 days post-viral inoculation (dpvi) in both wild-type (WT) and *TaBCAT1* double disruption mutant (TaBCAT1-A^{Q50*} TaBCAT1-B^{R366-}) plants. *TaASIL1* and *TaASIL2* expression levels were measured by RT-qPCR in plants silenced with *BSMV::TaASIL1* or *BSMV::TaASIL2* and compared to the negative control; *BSMV::msc4D* was used as a viral infection control. Three samples each were evaluated for *BSMV::TaASIL1*, *BSMV::TaASIL2*, and *BSMV::msc4D* in each plant background. **E-G.** Silencing of *TaASIL2* in WT plants significantly increased *TaSIZ1* expression (**E**) and significantly decreased *PR1* (**F**) and *PR3* (**G**) expression, whereas silencing of *TaASIL2* in the *TaBCAT1* double disruption mutant significantly reduced *TaSIZ1* (**E**), *PR1* (**F**), and *PR3* (**G**) expression. Asterisks denote statistically significant differences between each pair of conditions (***: $p < 0.001$, *: $p < 0.05$; two-tailed *t*-test). KD, silencing. Bar represents median value, box signifies the upper (Q3) and lower (Q1) quartiles, and whiskers are located at 1.5 times the interquartile range.

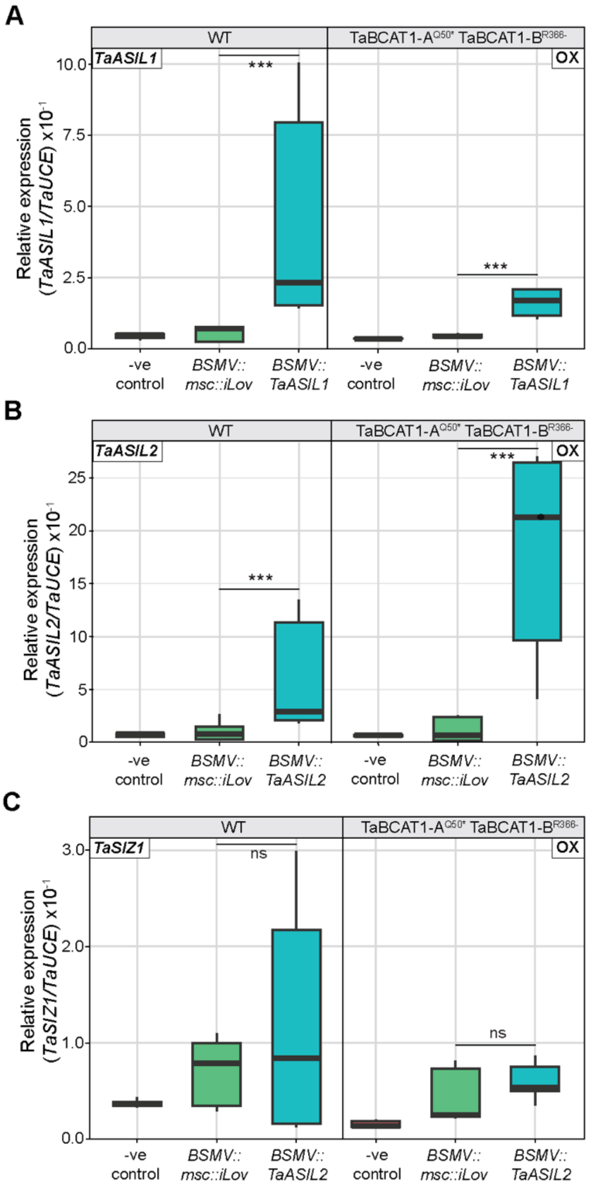

Supplemental Figure 5: Virus-induced overexpression of *TaASIL1* and *TaASIL2*. (Supports Figure 6.) **A-B.** *TaASIL1* and *TaASIL2* expression was significantly elevated 8–10 days post-viral inoculation (dpvi) in both wild-type (WT) and *TaBCAT1* double disruption mutant (TaBCAT1-A^{Q50*} TaBCAT1-B^{R366-}) plants. **C.** Overexpression of *TaASIL2* in both WT plants and the *TaBCAT1* double mutant led to no significant change in *TaSIZ1* expression. *TaASIL1* and *TaASIL2* or *TaSIZ1* expression levels were measured by RT-qPCR on plants inoculated with *BSMV::TaASIL1* (n = 3) or *BSMV::TaASIL2* (n = 3) and compared to the *BSMV::msc::iLov* positive fluorescent protein control (n = 3). Asterisks denote statistically significant differences between each pair of conditions (***: $p < 0.001$; two-tailed t-test). OX, overexpression. Bar represents median value, box signifies the upper (Q3) and lower (Q1) quartiles, and whiskers are located at 1.5 times the interquartile range.

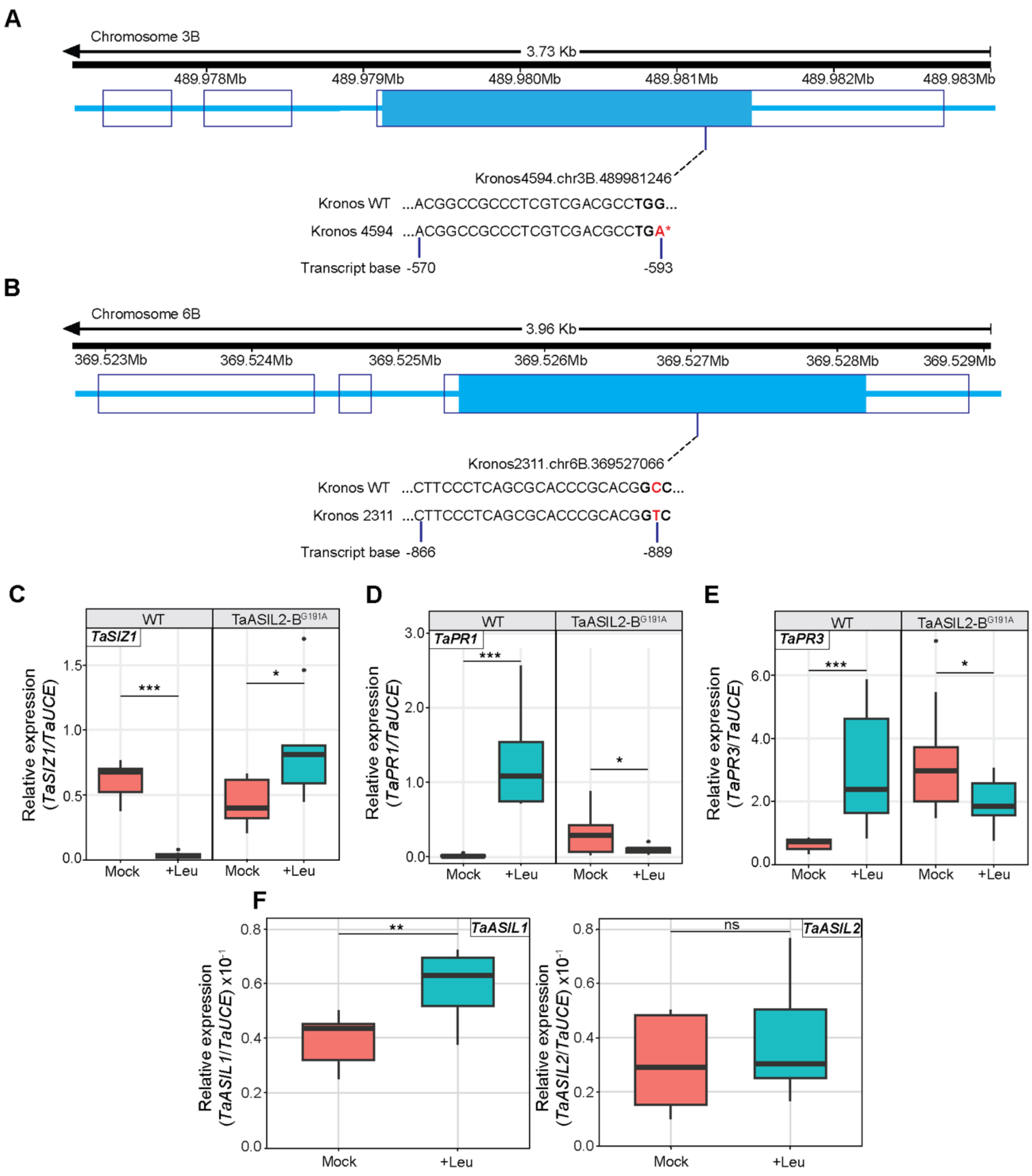

Supplemental Figure 6: Disrupting *TaASIL2* reduces the responses of *TaSIZ1*, *TaPR1*, and *TaPR3* to Leu treatment. (Supports Figure 6.) **A-B.** *TaASIL1* and *TaASIL2* are located on chromosomes 3 and 6 respectively, with loss-of-function tetraploid Kronos TILLING (Targeting Induced Local Lesions in Genomes) mutants identified for each gene in the B genome. For *TaASIL1*, a B genome mutant (Kronos4594) was selected that harbours an early stop codon mutation at amino acid 31, and for *TaASIL2*, a B genome mutant was selected (Kronos 2311) with a missense variant at amino acid 191 that converts Gly to Ala. Chr, chromosome; kb, kilobase; WT, wild-type. **C-E.** The *TaASIL2* disruption mutant (TaASIL2-B^{G191A}) displayed a minor increase in *TaSIZ1* (**C**) expression and decline in *PR1* (**D**) or *PR3* (**E**) gene expression following Leu treatment. By contrast, WT plants displayed a significant reduction in *TaSIZ1* (**C**) expression and increases in *TaPR1* (**D**) and *TaPR3* (**E**) expression following Leu treatment. **F.** Leu treatment (400 mg/L) led to an increase in *TaASIL1* expression, with no change detected in *TaASIL2* expression. RT-qPCR was conducted on three samples for each wheat line and treatment. Asterisks denote statistically significant

differences between each pair of conditions (***: $p < 0.001$, **: $p < 0.01$, *: $p < 0.05$; two-tailed *t*-test). Bar represents median value, box signifies the upper (Q3) and lower (Q1) quartiles, and whiskers are located at 1.5 times the interquartile range.

Supplemental Table S1: Details of the 15 predicted transcription factors that were enriched in the *TaBCAT1* disruption mutant compared to the wild-type. The results of nuclear proteomic analyses are shown. The abundance ratio ("Ratio") reflects differential abundance when comparing datasets between the *TaBCAT1* double disruption mutant (TaBCAT1-A^{Q50*} TaBCAT1-B^{R366-}) and wild-type plants.

UniProt IDs	Ratio	Protein Names	Transcription factor Family
A0A1D5UPA4	1.114	AT-hook motif nuclear-localized protein	AT-HOOK MOTIF NUCLEAR LOCALIZED (AHL)
A0A3B6B9B9	0.945	AT-hook motif nuclear-localized protein	AT-HOOK MOTIF NUCLEAR LOCALIZED (AHL)
A0A3B6REE4	0.917	AT-hook motif nuclear-localized protein	AT-HOOK MOTIF NUCLEAR LOCALIZED (AHL)
A0A3B5Z548	0.847	Homeobox domain-containing protein	Homeobox and DDT domain-containing transcriptional regulators
A0A3B6EH24	0.746	Homeobox domain-containing protein	Homeobox and DDT domain-containing transcriptional regulators
A0A3B6FS38	0.548	Homeobox domain-containing protein	Homeobox and DDT domain-containing transcriptional regulators
A0A3B6SDE0	1.198	Transcription factor ranscription factor CBF/NF-Y/archaeal histone domain-containing protein	Nuclear factor-Y (NF-Y) transcription factors
A0A3B6HY69	0.811	Transcription factor CBF/NF-Y/archaeal histone domain-containing protein	Nuclear factor-Y (NF-Y) transcription factors
A0A3B6LY30	0.641	Transcription factor CBF/NF-Y/archaeal histone domain-containing protein	Nuclear factor-Y (NF-Y) transcription factors
A0A077RZA1	1.713	MYB/SANT-like DNA-binding domain-containing protein	myeloblastosis viral oncogene homolog (MYB) family
A0A3B6NVP9	1.425	Myb/SANT-like domain-containing protein	myeloblastosis viral oncogene homolog (MYB) family
A0A3B6PN54	1.306	MYB/SANT-like DNA-binding domain-containing protein	myeloblastosis viral oncogene homolog (MYB) family
A0A3B6PST6	1.053	MYB/SANT-like domain-containing protein	myeloblastosis viral oncogene homolog (MYB) family
A0A3B6E9S7	0.978	MYB-like domain-containing protein	myeloblastosis viral oncogene homolog (MYB) family
A0A077RT14	0.9	MYB-like domain-containing protein	myeloblastosis viral oncogene homolog (MYB) family

Supplemental Table S2: RT-qPCR primers and efficiencies.

Gene name	Gene ID	Forward (5' – 3')	Reverse (5' – 3')	Efficiency	Reference
TaSIZ1	TraesCS1A02G065700 & TraesCS1B02G083900	TCGGAGGTGGAGATACTGC	GACCGCTGATTCAGTTCCA	96.63%	This study
TaUCE (reference)	TraesCS4A01G414200	CGGGCCCGAAGAGAGTCT	ATTAACGAAACCAATCGACGGA	97.10%	(Borrill et al., 2016)
TaPR1	TraesCS5A02G183300	CTGGAGCACGAAGCTGCAG	CGAGTGCTGGAGCTTGCAGT	102.21%	(Corredor-Moreno et al., 2021)
TaPR3	TraesCS1A02G203700	AGAGATAAGCAAGGCCACGTC	GGTTGCTCACCAGGTCCTTC	98.05%	(Corredor-Moreno et al., 2021)
TaASIL1	TraesCS3A02G271400 & TraesCS3B02G305100	GCGGCAACAAAAGGAAGAGG	GTTGAAGCTGCATGTCCACG	95.80%	This study
TaASIL2	TraesCS6A02G198600 & TraesCS6B02G230000	GTAATTCAGCCTCTACAGCA	CCAGAATTGCCCAGGAGCTT	101.76%	This study
PstEF1	PstEF1	TCGTGTCGAAACCGGTACCATCAA	AAACCAACGTTGTCACCTGGCAT	96.63%	(Tao et al. 2020)

Supplemental Table S3: Primers used for promoter and gene cloning for yeast one-hybrid assays. Lowercase, restriction enzyme recognition site extensions.

Region	Target species	Gene ID	Forward (5' – 3')	Reverse (5' – 3')	Reference
AtSIZ1 promoter	*A. thaliana*	AT5G60410	aacccggg ATATGTAACATGGGTCACAAAGG	aaacgcgt GACCGTAACGGAGTCTT	This study
TaSIZ1 promoter	*T. aestivum*	TraesCS1A02G065700 & TraesCS1B02G083900	aacccggg ATGGAAAGCTCGTAGGCAATGG	aaacgcgt CGTTTTGTACCGACGTGGTGCT	This study
TaPKS1-A promoter	*T. aestivum*	TraesCS2A02G267200	aagaattc CGGTAAAGAAAAATCAGCCGGTT	aactcgag TCCCTTTTCTTCTTTTTCTTCTTTCTT	(Zhang et al., 2024)
TaNF1 gene	*T. aestivum*	TraesCS6B02G316700	aagaattc ATGGGAGGGAGCAGCAAGAA	aactcgag CTAATATCCTTCCGGCGGC	(Zhang et al., 2024)

Supplemental Table S4: KASP primers used to genotype selected TILLING lines. Primers were designed with additional standard FAM or HEX compatible tails. Wild-type (WT) primers (A) were designed with FAM tails (5′-GAAGGTGACCAAGTTCATGCT-3′) and MUT primers (B) with HEX tails (5′-GAAGGTCGGAGTCAACGGATT-3′).

Gene name	Gene ID	Line	WT primer (A)	MUT primer (B)	Common primer
TaASIL1-B	TraesCS3B02G305100	Kronos4594	gccgccctcgtcgacgcctg**G**	gccgccctcgtcgacgcctg**A**	gggtcgcgatacatcgacct
TaASIL2-B	TraesCS6B02G230000	Kronos2311	cgcacccgcacgg**C**	cgcacccgcacgg**T**	tgtcaacttccctcagcgc

REFERENCES

Borrill, P., Ramirez-Gonzalez, R., and Uauy, C. (2016). expVIP: a Customizable RNA-seq Data Analysis and Visualization Platform. Plant Physiology **170,** 2172-2186.

Corredor-Moreno, P., Minter, F., Davey, P.E., Wegel, E., Kular, B., Brett, P., Lewis, C.M., Morgan, Y.M.L., Macias Perez, L.A., Korolev, A.V., et al. (2021). The branched-chain amino acid aminotransferase TaBCAT1 modulates amino acid metabolism and positively regulates wheat rust susceptibility. Plant Cell **33,** 1728-1747.

Lee, W.S., Rudd, J.J., and Kanyuka, K. (2015). Virus induced gene silencing (VIGS) for functional analysis of wheat genes involved in *Zymoseptoria tritici* susceptibility and resistance. Fungal Genet Biol **79,** 84-88.

Zhang, P.P., Guo, L.J., Long, J.Y., Chen, T., Gao, W.D., Zhang, X.F., Ma, J.F., Wang, P., and Yang, D.L. (2024). Genome-wide identification of phytosulfokine (PSK) peptide family reveals *TaPSK1* gene involved in grain development of wheat (*Triticum aestivum* L.). Chem Biol Technol Ag **11**.
